# Hyperbaric Oxygen Activates Enzyme‐Driven Cascade Reactions for Cooperative Cancer Therapy and Cancer Stem Cells Elimination

**DOI:** 10.1002/advs.202301278

**Published:** 2023-04-28

**Authors:** Yuxuan Xiong, Zhengtao Yong, Chen Xu, Qingyuan Deng, Qiang Wang, Shiyou Li, Chong Wang, Zhijie Zhang, Xiangliang Yang, Zifu Li

**Affiliations:** ^1^ National Engineering Research Center for Nanomedicine College of Life Science and Technology Huazhong University of Science and Technology Wuhan 430074 P. R. China; ^2^ Key Laboratory of Molecular Biophysics of Ministry of Education College of Life Science and Technology Huazhong University of Science and Technology Wuhan 430074 P. R. China; ^3^ Hubei Key Laboratory of Bioinorganic Chemistry and Materia Medical Huazhong University of Science and Technology Wuhan 430074 P. R. China; ^4^ Hubei Engineering Research Center for Biomaterials and Medical Protective Materials Huazhong University of Science and Technology Wuhan 430074 P. R. China; ^5^ Hubei Bioinformatics and Molecular Imaging Key Laboratory College of Life Science and Technology Huazhong University of Science and Technology Wuhan 430074 P. R. China; ^6^ GBA Research Innovation Institute for Nanotechnology Guangdong 510530 P. R. China

**Keywords:** cancer stem cells, chemodynamic therapy, combination therapy, hyperbaric oxygen, starvation therapy

## Abstract

Tumor starvation induced by intratumor glucose depletion emerges as a promising strategy for anticancer therapy. However, its antitumor potencies are severely compromised by intrinsic tumor hypoxia, low delivery efficiencies, and undesired off‐target toxicity. Herein, a multifunctional cascade bioreactor (HCG), based on the self‐assembly of pH‐responsive hydroxyethyl starch prodrugs, copper ions, and glucose oxidase (GOD), is engineered, empowered by hyperbaric oxygen (HBO) for efficient cooperative therapy against aggressive breast cancers. Once internalized by tumor cells, HCG undergoes disassembly and releases cargoes in response to acidic tumor microenvironment. Subsequently, HBO activates GOD‐catalyzed oxidation of glucose to H_2_O_2_ and gluconic acid by ameliorating tumor hypoxia, fueling copper‐catalyzed •OH generation and pH‐responsive drug release. Meanwhile, HBO degrades dense tumor extracellular matrix, promoting tumor accumulation and penetration of HCG. Moreover, along with the consumption of glucose and the redox reaction of copper ions, the antioxidant capacity of tumor cells is markedly reduced, collectively boosting oxidative stress. As a result, the combination of HCG and HBO can not only remarkably suppress the growth of orthotopic breast tumors but also restrain pulmonary metastases by inhibiting cancer stem cells. Considering the clinical accessibility of HBO, this combined strategy holds significant translational potentials for GOD‐based therapies.

## Graphical abstract

A versatile cascade bioreactor is developed to achieve an outstanding synergistic starvation‐/chemodynamic‐/chemo‐therapy with the help of hyperbaric oxygen (HBO). The amplified oxidative stress and HBO‐mediated tumor extracellular matrix degradation enable effective elimination of cancer stem cells, suppressing both orthotopic tumor and pulmonary metastasis.

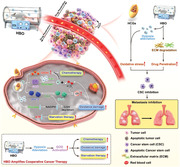

## 1 Introduction

Hypoxia, an important hallmark of tumor microenvironment (TME) for solid tumors, is caused by cancer cell fast proliferation and blood vessel abnormality during tumor angiogenesis.^[^ ^1^ ^]^ Mounting studies have confirmed that tumor hypoxia is responsible for resistance of numerous therapeutic treatments, including chemotherapy,^[^ ^2^ ^]^ radiotherapy,^[^ ^3^ ^]^ and photodynamic therapy.^[^ ^4^ ^]^ Additionally, reports show that hypoxia and hypoxia‐inducible factors (e.g., HIF‐1 and HIF‐2) expression are tightly associated with increased population of cancer stem cells (CSCs). Although accounting for only a small fraction of cancer cells in a hypoxic niche distal from the tumor vasculature, CSCs play a key role in tumor metastasis, which is the leading cause of death in cancer patients.^[^ ^5^ ^]^ Hence, allaying tumor hypoxia is crucial for the successful treatment of malignant tumors. At present, a variety of attempts have been devoted to oxygenate TME for achieving satisfactory therapeutic outcomes. For instance, perfluorocarbons^[^ ^6^ ^]^ or hemoglobin^[^ ^7^ ^]^ are frequently employed as oxygen carriers to transport molecular oxygen directly to tumor sites. Unfortunately, the inevitable leakage of oxygen during the circulation process will discount oxygen delivery efficiency.^[^ ^8^ ^]^ Another popular strategy is to catalyze the decomposition of endogenous H_2_O_2_ by catalase^[^ ^9^ ^]^ or metal‐based nanoparticles^[^ ^10^ ^]^ to generate oxygen in situ. However, limited H_2_O_2_ (0.5 nmol/10^4^ tumor cells h^−1^) in tumor tissues is insufficient to support continuous O_2_ generation in situ.^[^ ^11^ ^]^ Alternatively, hyperbaric oxygen (HBO) therapy, as a widely used clinical treatment, has been proven to ameliorate hypoxia in solid tumors by increasing the amount of dissolved oxygen in the plasma.^[^ ^12^ ^]^ In light of the safety, accessibility, and effectiveness of HBO therapy, the combination of HBO with oxygen‐dependent tumor therapeutic strategies shows great promise for clinical applications.

Recently, starvation therapy, represented by glucose oxidase (GOD), has been recognized as a feasible strategy to fight cancer by cutting off the necessary nutrients to tumors.^[^ ^13^ ^]^ As a natural aerobic dehydrogenase, GOD can consume intracellular glucose by oxidative decomposing glucose into gluconic acid and H_2_O_2_ to achieve tumor starvation effect.^[^ ^14^ ^]^ During this oxidation process, large amounts of H_2_O_2_ and H^+^ are generated, benefiting therapeutic strategies that depend on an adequate H_2_O_2_ supply or an acidic microenvironment.^[^ ^15^ ^]^ Although such GOD‐driven cascade reaction is attractive for tumor therapy, contradictorily, TME is often hypoxic, which greatly limits GOD‐mediated glucose oxidation and subsequent cascade reactions.^[^ ^16^ ^]^ More troublesome, the oxygen consumption arising from GOD oxidation will further aggravate tumor hypoxia, which is not only detrimental to GOD‐driven tumor therapy but also will increase the percentage of CSCs and tumor metastasis.^[^ ^5^, ^17^ ^]^ In addition, as a therapeutic protein, GOD is often plagued by innate drawbacks, such as poor stability, short circulation time, and off‐target catalytic activity and toxicity.^[^ ^14^, ^18^ ^]^ Leveraging efforts from HBO and nanomedicines, we envision that the GOD‐based therapeutic nanomedicine can achieve robust antitumor therapy with the help of HBO.

**Scheme 1:**
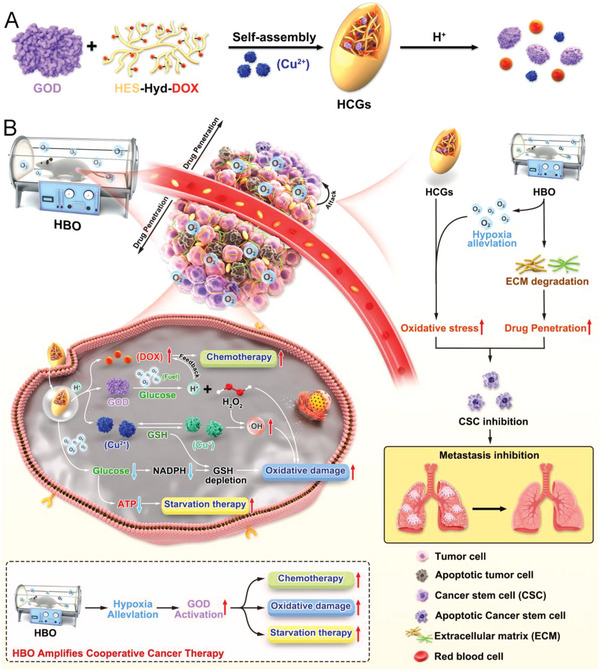
Schematic illustration of A) the fabrication process of HCG and B) the therapeutic mechanism of HBO‐activated HCG‐triggered cooperative cancer therapy.

In the present work, a pH‐sensitive detachable bioreactor (denoted as HCG) integrated with hydrazone‐bond‐linked hydroxyethyl starch (HES, a blood plasma volume expander for clinical use) and doxorubicin conjugate (HPD), copper ions (Cu^2+^) and GOD, is constructed via one‐step self‐assembly for tumor growth and metastasis inhibition under the help of HBO (**Scheme** **1**). In this bioreactor, HPD, as the framework of HCG, enables the acid‐responsive drug release and behaves as stabilizer for this nanoplatform. The Cu^2+^ ions in HCG can be efficiently reduced to Cu^+^ by the overexpressed glutathione (GSH) in tumor cells, subsequently catalyzing intracellular H_2_O_2_ to generate highly toxic •OH for effective chemodynamic therapy (CDT).^[^ ^19^ ^]^ Importantly, GOD‐catalyzed oxidation of glucose to produce H_2_O_2_ and gluconic acid not only starves tumor cells but also provides H_2_O_2_ for Cu^+^‐mediated CDT and lowers tumor pH to promote acid‐responsive release of doxorubicin (DOX) from HPD. As an enabler, HBO not only surmounts the inherent hypoxic microenvironment of tumors to activate GOD‐driven therapeutic nanoplatform, but more importantly degrades the dense tumor extracellular matrix (ECM) to promote the exposure of HCG to CSCs. Meanwhile, during the therapeutic process, the redox reaction of Cu^2+^ ions and the consumption of glucose by GOD are accompanied by the attenuation of antioxidant ability in tumors, further amplifying CDT‐induced oxidative stress. By integrating HBO‐boosted starvation‐/chemodynamic‐/chemo‐therapy, HBO‐mediated ECM degradation, and redox homeostasis disruption together, the fabricated HCG exhibits a remarkable therapeutic effect against both bulk cancer cells and intractable CSCs, inhibiting both orthotopic tumor and distant metastasis (Scheme 1).

## 2 Results and Discussion

### 2.1 Preparation and Characterization of HCG

**Figure 1:**
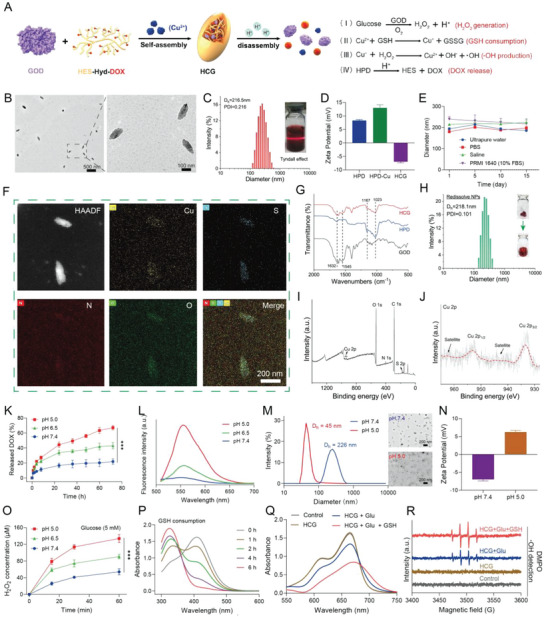
Characterizations of HCG. A) The synthesis diagram and action mechanism of HCG. B) TEM images of HCG at different magnifications. The scale bars for low and high magnification are 500 and 100 nm, respectively. C) DLS size distribution of HCG. D) Zeta potentials of HPD, HPD‐Cu, and HCG. E) Stability assessment of HCG in different media. F) Elemental mappings of HCG. Scale bars: 200 nm. G) FT‐IR spectra of GOD, HPD, and HCG. H) DLS size distribution of redissolved HCG from lyophilization. I) Survey XPS spectrum, and J) high‐resolution Cu 2p spectrum of HCG. K) DOX release profiles under different pH conditions. L) Fluorescence intensity of DOX after incubation at different pH conditions. M) The size (scale bars: 200 nm) and N) zeta potential changes of HCG after incubation in acidic condition. O) The ability of HCG to produce H_2_O_2_ in the presence of glucose after incubation at different pH conditions. P) GSH consumption ability of HCG. Q) MB degradation with different treatments. R) ESR spectra of **•**OH trapped by DMPO. Statistical significance was calculated by *t*‐test. *p* values, *** *p* < 0.001.

Initially, the pH‐responsive prodrug HES‐Hyd‐DOX (HPD) was synthesized by a triple‐steps process using an acid‐labile hydrazone bond as linker (Figure S1, Supporting Information).^[^ ^20^ ^]^ The successful conjugate of DOX with HES was confirmed by ^1^H NMR (Figure S2, Supporting Information). Subsequently, HCG was prepared by a simple self‐assembly method leveraging coordination, hydrophobic, and electrostatic interactions. The morphological structure of HCG was detected by transmission electron microscopy (TEM). As clearly shown in **Figure** **1**B, HCG exhibited a fusiform nanostructure with a length of 183 nm and width of 79 nm. Meanwhile, the average hydrodynamic diameter of HCG is around 216.5 nm (Figure 1C), as determined by dynamic light scattering (DLS). A digital photo of HCG dispersed in water with typical Tyndall effect indicated their excellent hydrophilicity and dispersity. The zeta potential of HCG was −7.0 mV, which is sharply different from that of HPD‐Cu (+13.1 mV) (Figure 1D). The difference in zeta potential between HCG and HPD‐Cu is probably because the negatively charged GOD neutralizes the positive charge of HPD‐Cu.^[^ ^21^ ^]^ This phenomenon also confirms the electrostatic interactions between GOD and HPD‐Cu. Furthermore, the stability of HCG in physiological environment was measured by DLS, and the results (Figure 1E) showed that HCG remained stable within 15 days, which satisfied the demands for subsequent biological experiments. Such excellent stability may be attributed to the shielding effect of HES.^[^ ^22^ ^]^ More importantly, the lyophilized HCG can be immediately redissolved in phosphate‐buffered saline with negligible size change (Figure 1H), greatly facilitating its long‐term storage, which is vital for clinical translation.

Fourier transform infrared spectrum (FT‐IR) characterizations were performed to determine the chemical structure of HCG. Concretely, the characteristic peaks at 1023 and 1167 cm^−1^ were attributed to the C—O stretching vibration in HPD.^[^ ^23^ ^]^ The peaks at 1545 cm^−1^ (amide II) and 1632 cm^−1^ (amide I)were assigned to amide groups of GOD (Figure 1G).^[^ ^24^ ^]^ These results demonstrated the successful integration of GOD into HCG. Notably, the redshift of the absorption peak of HCG was accompanied by the decrease of absorption at 485 nm and the enhancement of absorption at 580 nm, indicating the involvement of Cu in the coordination reaction with DOX (Figure S3, Supporting Information).^[^ ^25^ ^]^ In the meantime, the loading content of DOX was calculated to be about 6.5% in HCG as determined by UV‐vis measurements. Furthermore, energy dispersive X‐ray spectroscopy mapping images (Figure 1F) indicated that Cu, S, O, and N were homogenously distributed in HCG. Of these elements, the distribution of S was attributed to GOD, which further verified the successful incorporation of GOD into HCG. Moreover, the X‐ray photoelectron spectroscopy (XPS) was conducted to study the chemical composition of HCG. The XPS survey spectra (Figure 1I) verified the existence of Cu, S, O, and N in HCG. The high‐resolution spectra of Cu 2p (Figure 1J) showed two characteristic peaks at 933.5 eV (Cu 2p_3/2_) and 953.3 eV (Cu 2p_1/2_) accompanied by two shake‐up satellite peaks (942.1 and 962.1 eV), which were indexed to Cu^2+^.^[^ ^26^ ^]^ Moreover, the Cu^2+^‐doping ratio of HCG was determined to 1.58% by inductively coupled plasma optical emission spectroscopy (ICP‐OES).

Since there were numerous pH‐responsive hydrazone bonds in HCG, we then carefully investigated the pH‐responsive drug release behavior of HCG. As exhibited in Figure 1K, only 21.8% of DOX was liberated from HCG after 72 h of incubation under physiological conditions (pH 7.4, 37 °C), while the release of DOX can reach 43% at pH 6.5. In sharp contrast, the DOX release was accelerated under the condition of pH 5.0, with maximum DOX release up to 70%. The excellent acidic responsiveness of HCG was also confirmed by fluorescence spectrum at different pH values (Figure 1L). Considering that acid‐responsive HPD prodrugs represented the main structure of HCG, it was expected that the breakage of hydrazone bonds in HPD may also accelerate the structural disassembly and degradation of HCG. To test this hypothesis, HCG was immersed into buffer solutions with different pH to evaluate the disassembly of HCG. As depicted in Figure 1M, the particle size and morphology of HCG did not change significantly after 72 h incubation at pH 7.4. However, the particle size of HCG was obviously decreased (*D*_h_ = 45 nm) when incubated at an acidic condition (pH 5.0), which was also evidenced by the TEM images. These smaller nanoparticles seem to be hydroxyethyl starch drug conjugates.^[^ ^27^ ^]^ Unlike the degradation characteristics in acidic microenvironments, HCG remained intact in glucose solution (Figure S4, Supporting Information). Such sustained endogenous stimulation‐triggered nanomedicines are beneficial in reducing off‐target effects and improving long‐term antitumor efficiency. Interestingly, the zeta potential of HCG reverses from −7 to +6.2 mV under acidic conditions, probably due to the shedding of negatively charged GOD. More attractively, we found that the catalytic activity of GOD in HCG exhibited acid response features. The catalytic activity of GOD was estimated by H_2_O_2_ production. As shown in Figure 1O, compared to preincubation at pH 7.4 buffer, HCG preincubated in acidic buffer (pH 6.5, pH 5.0) yielded more H_2_O_2_ in the presence of glucose (5 × 10^−3^ m). Notably, the H_2_O_2_ produced after preincubation at pH 5.0 condition was 2.5 times higher than that at pH 7.4. This acidic‐enhanced GOD catalytic activity may be due to 1) the formation of HCG impeded the catalytic reaction of GOD with the substrate, and 2) the binding of hydrophobic DOX molecules with the hydrophobic domain of GOD inhibited the catalytic activity of GOD.^[^ ^18b^ ^]^ Therefore, the disassembly of HCG under acidic conditions restored the catalytic activity of GOD. In view of the presence of glucose in blood, this acid‐responsive characteristic of HCG may improve its biosafety during blood circulation, which was also beneficial for tumor‐specific therapy.

The redox nature of Cu^2+^ motivated us to investigate the ability of HCG in GSH consumption. As expected, the GSH (1 × 10^−3^ m) was completely consumed in the reaction mixture after 6 h incubation (Figure 1P). After reaction with GSH, Cu^2+^ in HCG was reduced to Cu^+^, which can catalyze the production of •OH with H_2_O_2_.^[^ ^19a^ ^]^ We have proved above that under acidic conditions, HCG can produce substantial amounts of H_2_O_2_ in the presence of glucose, which was expected to provide sustained H_2_O_2_ for Cu^+^‐mediated Fenton‐like reaction (Figure 1A). Hence, we further employed methylene blue (MB) as a probe^[^ ^28^ ^]^ to evaluate the generation of •OH by HCG. Specifically, after incubation with HCG, the absorbance of MB was not obviously changed in the absence of glucose. When glucose (5 × 10^−3^ m) was added, a pronounced MB degradation could be observed, indicating the generation of •OH. This result demonstrated that glucose was essential for HCG to generate •OH. In comparison, HCG that incubated with glucose (5 × 10^−3^ m) and GSH (1 × 10^−3^ m) exhibited more intense MB degradation, suggesting that Cu^2+^ was reduced to Cu^+^ by GSH, which then catalyzed more •OH production (Figure 1Q). The generation of •OH was also examined by electron spin resonance (ESR) with 5,5‐dimethyl‐1‐pyrroline N‐oxide (DMPO) as spin trap agent.^[^ ^21^ ^]^ From the ESR spectra (Figure 1R), it was evident that the treatment of HCG + glucose + GSH induced strong •OH signal (peak intensity of 1:2:2:1). The results were in accordance with MB degradation experiments. Compared to normal cells, cancer cells often have higher GSH concentrations^[^ ^19b^ ^]^ and greater dependency on glucose.^[^ ^14a^ ^]^ Thus, the obtained HCG was expected to attain effective tumor‐specific therapy with the joint efforts of glucose depletion, reactive oxygen species (ROS) production, and acidity‐responsive DOX release (Figure 1A).

### 2.2 Cytotoxicity of HCG under Normoxic Conditions

**Figure 2:**
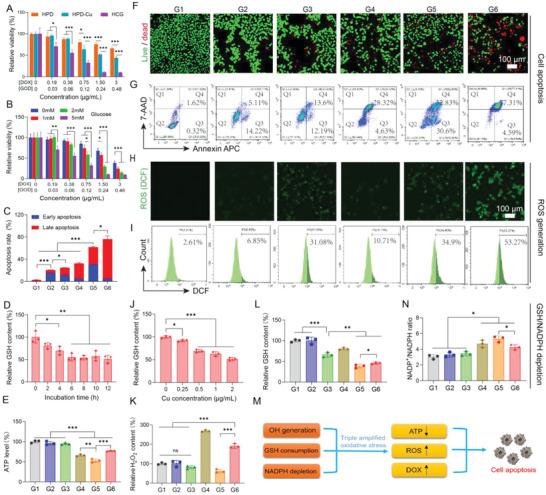
In vitro antitumor evaluation and mechanism study on 4T1 tumor cells in normoxic conditions. A) Cell viability assay of HPD, HPD‐Cu, and HCG on 4T1 cells. B) Cytotoxicity assessment of HCG under different glucose concentrations. C) Quantitation of cell apoptosis results measured by flow cytometry. D) The GSH levels of 4T1 cells treated with HCG for different times. E) ATP levels of 4T1 cells after various treatments. F) Live/dead staining (scale bars: 100 µm) and G) cell death mechanism of 4T1 cells after different treatments. ROS levels in 4T1 cells after different treatments identified by H) confocal imaging (scale bars: 100 µm) and I) flow cytometry. J) Relative GSH content in 4T1 cells after treatment with different concentrations of HCG. Relative K) H_2_O_2_, L) GSH, and N) NADP^+^/NADPH ratio contents in 4T1 cancer cells with various treatments. M) Summary of HCG‐induced cell apoptosis. Statistical significance was calculated by *t*‐test. *p* values: * *p* < 0.05, ** *p* < 0.01, *** *p* < 0.001, ns stands for not significant.

Based on the aforementioned properties of HCG, we further assessed the behaviors of HCG in 4T1 cancer cells. In view of the indispensability of O_2_ for GOD‐induced catalytic reactions, we investigated the cellular activity of HCG under normoxic conditions (21% O_2_). We first investigated the cellular uptake and lysosomal escape of HCG using confocal laser scanning microscopy (CLSM). The results demonstrated a time‐dependent internalization of HCG. Most HCGs were sequestered in lysosomes after endocytosis, and some of the green fluorescence (DOX) was transferred from the lysosome to the cytoplasm when the incubation time was extended to 8 h. As the incubation time reached 12 h, the DOX fluorescence in the cytoplasm was further intensified, indicating lysosome escape of HCG (Figure S5, Supporting Information). Moreover, the in vitro cytotoxicity of HCG was evaluated by standard 3‐[4,5‐dimethylthiazol‐2‐yl]‐2,5 diphenyl tetrazolium bromide (MTT) assay. As illustrated in **Figure** **2**A, the acidity‐responsive prodrug HPD showed moderate cytotoxicity, with 66.3% cell viability at DOX concentration of 3 µg mL^−1^. When Cu^2+^ was introduced via coordination, the viability of 4T1 cells decreased appreciably in a concentration‐dependent manner, which was attributed to Cu^2+^‐mediated CDT. In stark contrast, HCG exhibited dramatically higher cytotoxicity than HPD or HPD‐Cu at all set concentrations, confirming the powerful potentiation effect of GOD on this nanoplatform. Besides, as can be seen in Figure 1B, increasing either the glucose concentration or the HCG concentration can enhance cytotoxicity. Meanwhile, the lethality of HCG on normal cells was assessed (Figure S6, Supporting Information). MTT results showed that the half maximum inhibitory concentration (IC50) of NIH‐3T3 and HUVECs were 1.846 and 1.661 µg mL^−1^ (DOX), respectively, which was higher than the lethality of HCG on 4T1 cells (IC50 = 0.40 µg mL^−1^). The difference in cytotoxicity may be due to the specific microenvironment and higher glucose‐dependent nature of the tumor cells.^[^ ^14^, ^19^ ^]^ In order to observe live and dead cells visually, Calcein AM/7‐AAD double staining assay was conducted (Figure 2F). As expected, HCG incubated with glucose‐containing medium presented the highest percentage of dead cells, which was consistent with the results of MTT assay. Moreover, the lethal mechanism was analyzed by flow cytometry (Figure 2G). Compared with the control group, a certain degree of apoptosis and death ratio were observed in HPD‐treated group, attributed to the natural cytotoxicity of DOX. When Cu^2+^ was introduced, the degree of cell apoptosis was further increased, from 19.6% to 25.5%, thanks to copper‐mediated CDT. Free GOD caused a moderate degree of apoptosis, mainly ascribed to GOD‐induced tumor starvation and high H_2_O_2_ production. Besides, compared to incubation without glucose, HCG incubation in glucose‐containing medium produced markedly stronger cytotoxicity to 4T1 cells (61.7% vs 79.5%, Figure 2C), reflecting the contribution of GOD‐mediated glucose oxidation to the antitumor effect.

Given the results of in vitro cytotoxicity under normoxic conditions, we further studied the underlying mechanisms in detail. The intracellular ROS level was first assessed using the 2′,7′‐dichlorofluorescein diacetate (DCFH‐DA) probe.^[^ ^28^ ^]^ As presented in Figure 2H, weak green fluorescence was emitted after the cells treated with HPD or GOD alone, suggesting modest ROS was generated. Comparatively, stronger green fluorescence in the 4T1 cells was observed when cells were treated by HPD‐Cu, which was ascribed to Cu‐mediated ROS production. In striking contrast, 4T1 cells incubated with HCG in the presence of glucose presented the most intense green fluorescence (G6), but once glucose was withdrawn, the green fluorescence was reduced (G5). This phenomenon implied that GOD‐triggered glucose oxidation was conducive to intracellular ROS generation. Similar results were obtained by flow cytometry (Figure 2I). Previous literatures had revealed that excess GSH in tumor cells as an ROS scavenger would impair ROS production.^[^ ^19^, ^29^ ^]^ HCG‐induced profuse ROS production should also correlate with GSH consumption within tumor cells. To verify this hypothesis, we used GSH assay kit to characterize the consumption of intracellular GSH in different groups. The results in Figure 2D and 2J showed that HCG was able to consume intracellular GSH in a time‐ and concentration‐dependent manner. In comparison with HPD, both HPD‐Cu and HCG could markedly reduce GSH levels in 4T1 cells, indicating the doped Cu^2+^ had the ability to consume intracellular GSH^[^ ^19a^ ^]^ (Figure 2L). Interestingly, free GOD also exhibited some degree of GSH consumption, and stripping glucose from the medium would further diminish intracellular GSH, implying that glucose deprivation may also decrease GSH within cancer cells. It had been reported that glucose deprivation would limit NADPH production from the pentose phosphate pathway, while nicotinamide adenine dinucleotide phosphate (NADPH) provided reducing power for antioxidant agents such as GSH.^[^ ^30^ ^]^ We suspected that GOD‐induced glucose starvation should decrease GSH by inhibiting NADPH. Therefore, we further evaluated the effect of different treatments on NADPH. The NADP^+^/NADPH ratio was chosen as an indicator to evaluate NADPH level.^[^ ^31^ ^]^ As shown in Figure 2N, neither HPD nor HPD‐Cu caused significant changes in NADP^+^/NADPH ratio. However, the NADP^+^/NADPH ratio exhibited a significant increase in all groups containing GOD, in which 4T1 cells incubated with HCG (without glucose in medium) showed the highest NADP^+^/NADPH ratio. The blockage of NADPH would inhibit the reduction of GSSG to GSH, leading to the decrease in GSH content.^[^ ^19b^ ^]^ The above results confirmed that GOD‐triggered glucose deprivation can consume GSH by inhibiting NADPH. In addition, GOD‐induced glucose oxidation not only cut off energy supply and reduced intracellular adenosine‐5’‐triphosphate (ATP) supply, but also provided large amounts of H_2_O_2_ for Cu‐mediated Fenton‐like reaction. The ATP and H_2_O_2_ level in each group were evaluated afterward. As shown in Figure 2E, the HCG incubated with 4T1 cells in glucose‐free medium elicited the lowest ATP levels due to complete deprivation of glucose. Concurrently, free GOD treatment exhibited a lower ATP level and higher H_2_O_2_ production as compared to HCG, which may be attributed to the incomplete release of GOD from HCG (Figure 2K). With all the above observations, we may draw some conclusions about the mechanism of action for HCG‐induced apoptosis under normoxia. 1) The H_2_O_2_ produced by the catalytic oxidation of glucose fuels Cu^+^ to produce large amounts of toxic •OH. Meanwhile, the consumption of glucose and the redox reaction of Cu^2+^ weaken the antioxidant capacity of tumor cells. These two factors contribute to the massive accumulation of ROS in tumor cells. 2) HCG can be effectively taken by 4T1 cells to release DOX for chemotherapy. 3) GOD‐induced reduction of glucose causes tumor cell starvation by lowering intracellular ATP production. Thus, HCG induced oxidative damage, DOX release, and ATP depletion, together accounting for the effective therapeutic outcome of HCG (Figure 2M).

### 2.3 HBO Helps HCG Suppress Both Bulk Cancer Cells and CSCs in a Hypoxic Environment

**Figure 3:**
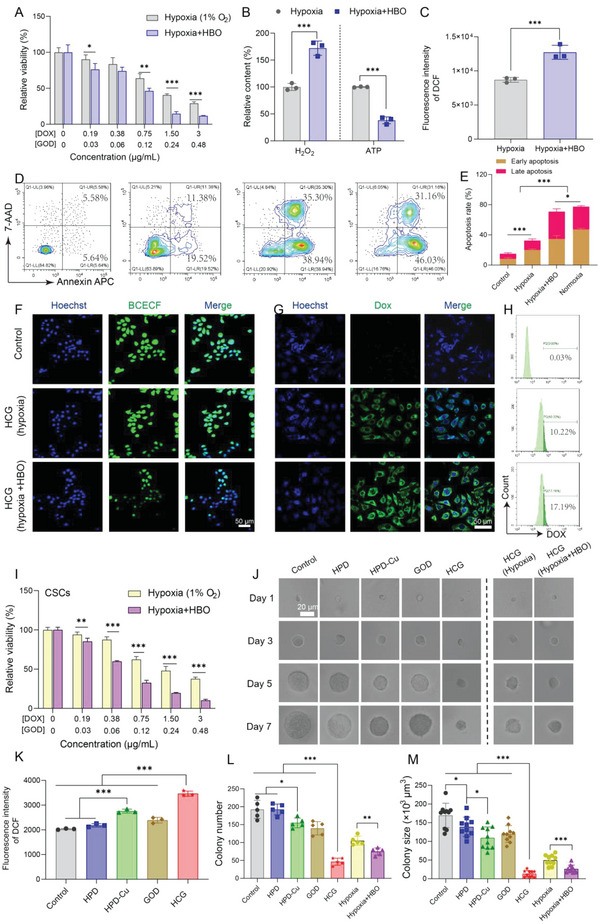
HBO helps HCG suppress both bulk cancer cells and CSCs under hypoxic conditions. A) Relative cell viability of 4T1 cells treated with HCG under hypoxia or hypoxia + HBO conditions. B) Relative H_2_O_2_ content, ATP level, and C) ROS level of HCG‐treated 4T1 cells incubated in hypoxia or hypoxia + HBO conditions. D,E) Analysis of apoptosis in 4T1 cells after different treatments by flow cytometry. F) CLSM images of 4T1 cells after different treatments and stained with pH fluorescent probe BCECF. Scale bars: 50 µm. G) CLSM images (scale bars: 50 µm) and H) flow cytometry analysis of DOX fluorescence in 4T1 cells after treated with HCG under hypoxia or hypoxia + HBO conditions. I) Relative viability of CSCs treated with HCG in hypoxia or hypoxia + HBO conditions. J) The spheroid growth of 4T1 cells seeded in soft 3D fibrin with different treatments. Scale bars: 20 µm. K) ROS level in CSCs after different treatments. L) The colony number and M) colony size at day 7. Statistical significance was calculated by *t*‐test. *p* values: * *p* < 0.05, ** *p* < 0.01, *** *p* < 0.001, ns stands for not significant.

Under normoxia condition, HCG showed excellent therapeutic efficacy against 4T1 cancer cells. But hypoxia is an intrinsic feature of the solid tumors,^[^ ^1a^ ^]^ which will inevitably weaken GOD‐triggered glucose oxidation and the subsequent cascade reactions. The HBO therapy is one of the most effective means of relieving hypoxia in solid tumors.^[^ ^32^ ^]^ The combination of HBO and HCG held promise for the treatment of hypoxic solid tumors. To verify the effect of this combination strategy, we first evaluated the GOD catalytic activity of HCG under different oxygen conditions. As illustrated in Figure S7 in the Supporting Information, the normoxic and HBO‐treated groups showed much higher H_2_O_2_ production than hypoxic treatment, confirming the essential role of oxygen for GOD‐triggered catalyzed reactions. Based on this result, we further investigated the cytotoxicity of HCG under different oxygen conditions. Under hypoxic condition, the cytotoxicity of HCG was significantly reduced compared to normoxic treatment, the IC50 increased from 0.40 µg mL^−1^ (DOX) to 1.32 µg mL^−1^ (DOX). When treated with HBO (2.5 ATA, 1.5 h) during the therapeutic process, the cell viability of HCG‐treated cells (IC50 = 0.62 µg mL^−1^) decreased dramatically relative to hypoxic incubation (**Figure** **3**A), due to the sufficient O_2_ supply by HBO. Additionally, the Annexin APC/7‐AAD‐based flow cytometry analysis yielded consistent results (Figure 3D,E) with the cytotoxicity assay described above. In the meantime, 4T1 cells in the HBO‐treated group exhibited a significant increase in H_2_O_2_ production (1.8 times higher than hypoxia group), and a dramatic decrease in ATP content (0.38 times lower than hypoxia) (Figure 3B). Compared to the hypoxic group, HBO did not significantly affect H_2_O_2_ production, but slightly increased ATP levels (Figure S8, Supporting Information). The increases of ATP levels were ascribed to the inhibition of mitochondrial respiration by hypoxia.^[^ ^33^ ^]^ These results indicated that HBO treatment could promote GOD‐mediated catalytic reactions and induce tumor cell starvation by supplying O_2_. Moreover, flow cytometric results (Figure 3C and Figure S9, Supporting Information) showed that the ROS level in HBO‐treated group was considerably higher than that in hypoxia group. This was probably owing to the activation of GOD by HBO, which drove the subsequent Cu‐mediated Fenton‐like reaction to produce •OH. It was worth noting that the GOD‐catalyzed oxidation of glucose could not only produce plentiful H_2_O_2_ for Fenton‐like reaction but also gluconic acid to increase acidity of TME.^[^ ^14a^ ^]^ Hence, we employed 2'‐7'‐bis(carboxyethyl)‐5(6)‐carboxyfluorescein (BCECF) as a pH probe to study the influences of HCG treatment on cellular pH under varying O_2_ atmospheres. As shown in Figure 3F, the green fluorescence in HBO‐treated group was obviously weaker than that in the hypoxia group, suggesting a decreased pH post HBO treatment. In view of the acidity‐responsive characteristics of HCG, the decrease in pH should facilitate the disassembly of HCG. As mentioned before, the disintegration of HCG under acidic conditions would lead to the recovery of DOX fluorescence (Figure 1L). Thus, we further assessed the release of DOX in cells after different treatments by measuring the fluorescence intensity of DOX. As observed in the CLSM images (Figure 3G), the HCG‐treated 4T1 cells with HBO exhibited much stronger green fluorescence than that of hypoxia group, indicating a higher DOX release in 4T1 cells. This result was further confirmed by flow cytometry analysis (Figure 3H). This phenomenon demonstrated that HBO treatment could promote the accumulation and release of DOX from HCG in hypoxic cancer cells. Taken together, the above results confirmed that HBO treatment could drive the catalytic oxidation of GOD to acidify tumor cells, which benefited DOX‐mediated chemotherapy.

Considering the crucial impact of CSCs on tumor initiation, progression, invasion, recurrence, and metastasis, killing the buck of differentiated cancer cells while eliminating CSCs was recognized as the key to successful cancer treatment.^[^ ^34^ ^]^ Compared to normal cancer cells, CSCs are more hypoxic because they are more distant from the chaotic tumor vasculature.^[^ ^35^ ^]^ Besides, regulating oxidative stress was deemed to be a viable therapeutic target for CSCs.^[^ ^36^ ^]^ We have demonstrated above that HCG can induce potent oxidative stress in 4T1 cells at sufficient oxygen supply conditions. Taking these into account, we envisaged that HCG may also have the capacity to eliminate CSCs. To substantiate this hypothesis, we first evaluated the cytotoxicity of breast CSCs (BCSCs) treated with different formulations via live/dead staining. The BCSCs were sorted on ultra‐low attachment plate as previously reported.^[^ ^12^, ^37^ ^]^ As shown in Figure S10 in the Supporting Information, exposure of BCSCs to HCG induced copious amounts of dead cells, while only a certain dead cell could be observed in the groups of HPD, HPD‐Cu, and GOD. Notably, the size of mammosphere cells reduced obviously after HCG treatment, probably because some of the dead tumor cells were separated from the mammospheres.^[^ ^38^ ^]^ It was reported that the higher antioxidant capacity of CSCs was one of the contributors to treatment resistance.^[^ ^36b^ ^]^ Hence, we further analyzed the ROS and GSH levels in BCSCs after treatment in each group. Compared with the other four groups, the BCSCs in HCG‐treated group exhibited a higher ROS accumulation (Figure 3K and Figure S11, Supporting Information) and a lower GSH content (Figure S12, Supporting Information), which may be one of the reasons for the excellent CSCs inhibition of HCG. Taking into account that CSCs normally resided in hypoxic regions away from the vasculature, we further quantitatively evaluated the killing effect of BCSCs by HCG in hypoxia or hypoxia + HBO conditions. Obviously, when HBO was implemented, cell survival of BCSCs incubated with HCG was significantly reduced compared to that of hypoxic condition (Figure 3I), with IC50 of 1.66 and 0.51 µg mL^−1^ (DOX) for hypoxia and hypoxia + HBO, respectively, proclaiming that HBO helps HCG effectively eliminate BCSCs. Besides culturing CSCs by ultra‐low attachment plate, we further adopted biomechanical approach to enrich CSCs by culturing single cancer cells in soft 3D fibrin gels (90 Pa). The formed spheroid colonies in fibrin gels were mainly composed of CSCs, which have been proven to have remarkable tumor propagation capacity.^[^ ^39^ ^]^ Based on this biomechanical approach, we evaluated tumor propagation capacity after different treatments by measuring the size and number changes of tumor spheroid colonies. As clearly imaged in Figure 3J, with the extension of culture time, the size of tumor spheroid colonies increased to different degrees, among which the growth rate of HCG‐treated group under normoxic condition was the slowest. On day 7, the number and size of tumor spheroid colonies in the different treatment groups were counted and measured. Under normoxic conditions, the tumor spheroid colonies in the HCG‐treated group were much lower than the other groups in terms of both colony number and size (Figure 3L,M), indicating that HCG could inhibit the propagation of CSCs to the greatest extent. However, the expansion of CSCs was significantly enhanced under hypoxia, probably because deprivation of oxygen silenced the catalytic cascade reaction of GOD in HCG. Nonetheless, when HBO (1.5 h, 2.5 ATA) was applied, the increased CSCs expansion from hypoxia was attenuated, with colony number decreased from 105 to 68 and the colony size diminished from 4.9 × 10^4^ to 2.6 × 10^4^ µm^3^ (Figure 3L,M). These results could be ascribed to the activation of GOD by HBO. These in vitro results proved that HCGs were effective in eradicating CSCs from tumor spheroids while HBO could help HCG inhibit the propagation of hypoxic CSCs.

### 2.4 HBO‐Enhanced Tumor Accumulation and Penetration

**Figure 4:**
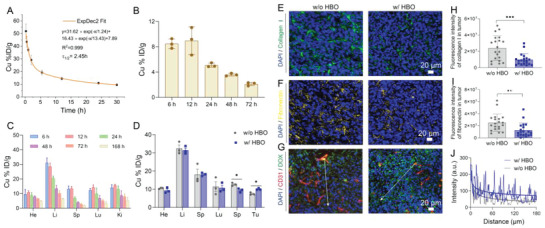
Pharmacokinetics study of HCG and HBO‐boosted tumor accumulation and penetration. A) The blood circulation curve of intravenously injected HCG (*n* = 3). B) The tumor accumulation of Cu in tumor at different times post‐intravenous administrations of HCG. C) Biodistribution of Cu in the main organs at different time points post‐injection of HCG. D) Biodistribution of Cu in main organs and tumor after intravenous injection of HCG with (w/) or without (w/o) HBO pretreatment. Immunofluorescence staining images of E) collagen I and F) fibronectin after different treatments. Scale bars: 20 µm. Corresponding quantitative analysis of H) collagen I and I) fibronectin staining. G) In vivo penetration of HCG in tumor tissues after different treatments. Scale bars: 20 µm. J) Relative Dox fluorescence intensity of a random line (shown in G) from tumor vessel to tumor interior. Line length is 180 µm, fluorescence intensity was analyzed with ImageJ. Statistical significance was calculated by *t*‐test. *p* values: * *p* < 0.05, ** *p* < 0.01, *** *p* < 0.001.

In light of the excellent results from above in vitro studies, HCG was applied on animal model in vivo. Hemolysis assay results show that there was no red cell lysis after incubation with different concentrations of HCG, and the hemolysis rate was almost no different from that of the negative control (Figure S13, Supporting Information). Even if the incubation time was extended to 12 h, the hemolysis rate was still lower than 10% (Figure S14, Supporting Information). These results indicated excellent compatibility of HCG with blood erythrocyte. Subsequently, the pharmacokinetic profile of HCG was studied to elucidate their blood circulation in vivo. As shown in **Figure** **4**A, the blood circulation half‐time of HCG was determined to be 2.45 h by a classical two‐compartment pharmacokinetic model. After systemic intravenous injection, the biodistribution analysis of HCG at various time intervals was further assessed. The relative distribution amounts of HCG within tumor were 8.4% in 6 h and reached the maximum (8.9%) at 12 h post‐injection (Figure 4B), which could be ascribed to the enhanced permeability and retention effect. To maximize HCG activation, 12 h after administration was identified as the optimal time for subsequent HBO treatment. Meanwhile, HCG was found to be abundantly distributed in the liver and spleen, mainly due to the uptake by the reticuloendothelial system. The retention of HCG in the major organs gradually decreased with time, and only a small amount of residual was left after 1 week (Figure 4C). Furthermore, we also assessed the influence of HBO treatment on the biodistribution of HCG. The results showed that HBO pretreatment could promote HCG tumor accumulation, with the relative content of HCG in tumor tissues increased from 7.6% to 10.1% (Figure 4D). This outcome may be explained by the fact that HBO treatment could degrade the dense ECM (Figure 4E,F,H,I and Figure S15, Supporting Information) and decompress the tumor blood vessels (Figure S16, Supporting Information). These results are highly consistent with our previous observations.^[^ ^12^, ^32^, ^40^ ^]^ Given that the barrier (ECM) to drug penetration was partly degraded by HBO, we then investigated the penetration of HCG in tumor tissues. As clearly illustrated in Figure 4G,J, HCG can reach deeper into tumor parenchyma after extravasation from tumor blood vessel following HBO treatment than that without HBO treatment. As CSCs tended to reside distal to tumor vessels, this HBO‐enhanced tumor penetration was favorable to HCG exposure to CSCs. Taken together, these results indicated that HCG exhibited a superior tumor accumulation while HBO could further promote HCG tumor enrichment and penetration.

### 2.5 In Vivo Antitumor Effect

**Figure 5:**
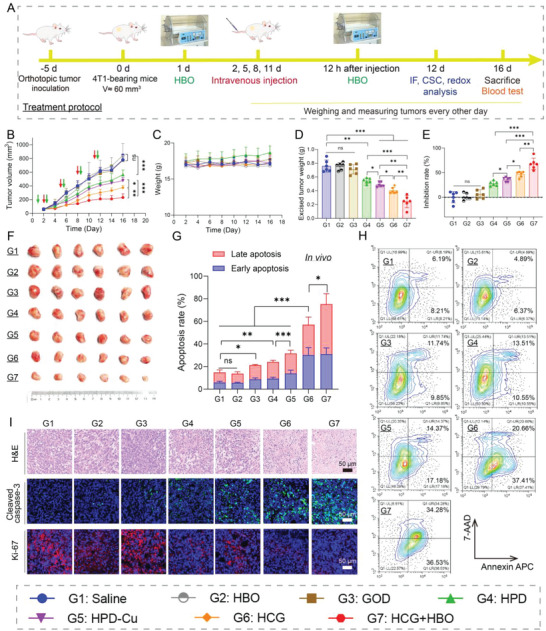
Pharmacodynamic evaluation in vivo. A) Schematic illustration of orthotopic 4T1 tumor model establishment and treatment protocol. B) Tumor volumes and C) body weights of the mice in various treatment groups. D) Average tumor weight at the end of different treatments. E) TIRs of different groups. F) Photographic images of tumors excised from different groups after various treatments. G,H) Assessment of apoptosis in tumor tissues after different treatments. I) H&E, Cleaved caspase‐3, and Ki‐67 staining images of tumor after different treatments. Scale bars: 50 µm. Statistical significance was calculated by *t*‐test. *p* values: * *p* < 0.05, ** *p* < 0.01, *** *p* < 0.001, ns stands for not significant.

Inspired by the satisfactory cytotoxicity and good tumor accumulation, we next investigated the antitumor performance of HCG on orthotopic 4T1 tumor model. When the tumor volume reached about 60 mm^3^, the tumor‐bearing mice were randomly grouped and subjected to different treatments (**Figure** **5**A). According to the results shown in Figure 4B, HBO was applied 12 h after intravenous injection of HCG. During therapies, the tumor volumes and body weights were recorded every other day. As displayed in Figure 5B, the tumor progression was markedly inhibited in mice injected with HCG in comparison to saline, HBO, GOD, HPD, and HPD‐Cu treated groups. When HBO was introduced, the tumor suppressive efficacy of HCG was further strengthened, with the tumor inhibition rate (TIR) increased from 46.7% to 68.0% (Figure 5E). The tumor weight (Figure 5D) and photographs of tumors (Figure 5F) excised from mice after various treatments also showed the highest tumor suppression in HCG+HBO treatment group. Meanwhile, no significant weight loss was observed throughout the treatment courses, indicating there was no acute toxicity during treatments (Figure 5C). The therapeutic outcome was also verified by histomorphology analysis. It can be seen in hematoxylin and eosin (H&E) staining that tumor tissues in HCG+HBO treated group exhibited the most obvious shrinking of the nuclei. Cleaved caspase‐3 staining showed the strongest green fluorescence in HCG+HBO‐treated group, indicating the maximal apoptosis of tumor cells. Moreover, the Ki67 staining revealed that there were much fewer proliferating tumor cells after HCG+HBO treatment compared to other groups (Figure 5I). To further gain insight into the therapeutic benefit in vivo, the cell apoptosis of tumor cells after different treatments was confirmed by flow cytometry. As anticipated, the HBO + HCG‐treated group had the highest percentage of apoptotic tumor cells, reaching 73.5%, which was significantly higher than other six groups (Figure 5G,H). Besides, no apparent abnormalities were observed in H&E staining of major organs (Figure S17, Supporting Information) and blood biochemical levels (Figures S18 and S19, Supporting Information), demonstrating the excellent biocompatibility of HCG and HBO combination therapy. These results collectively confirmed the excellent therapeutic performance of HCG assisted by HBO with minimal side effects.

### 2.6 In Vivo Mechanism Study

**Figure 6:**
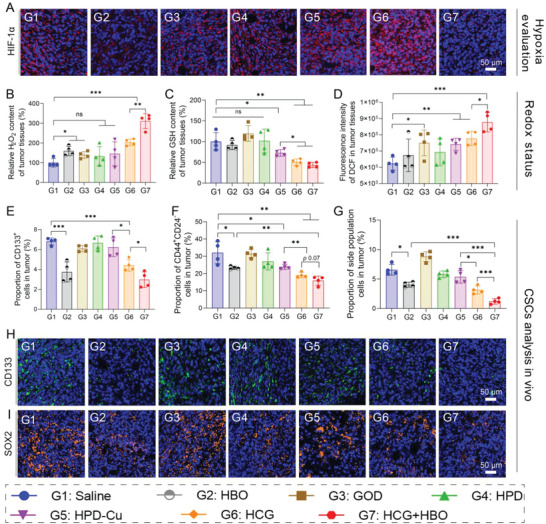
Hypoxia, redox, and CSC evaluation of tumor tissues after different treatments. A) HIF‐1*α* staining of tumor tissues retrieved from the mice post various treatments. Scale bars: 50 µm. B) H_2_O_2_ and C) GSH levels in tumor tissues of mice in different treatment groups tested by corresponding kits. D) ROS levels in tumor tissues after different treatments measured by flow cytometry. Percentage of CSCs in tumor tissues after different treatments characterized by flow cytometry and identified with E) CD133^+^, F) CD44^+^CD24^−^, and G) side population cells. Representative images of H) CD133 and I) SOX2 stained tumor tissues after different treatments. Scale bars: 50 µm. Statistical significance was calculated by *t*‐test. *p* values: * *p* < 0.05, ** *p* < 0.01, *** *p* < 0.001, ns stands for not significant.

To elucidate the therapeutic mechanism of HCG+HBO combination therapy, we further examined the key biomolecular indicators in tumor cells after various treatments. Considering that oxygen is a key factor affecting GOD‐mediated catalytic reactions, we first evaluated the hypoxic state of the tumors by HIF‐1*α* staining (**Figure** **6**A). Obviously, HBO treatment led to remarkable downregulation of HIF‐1*α*, indicating tumor hypoxia was successfully relieved. Without HBO, HCG treatment (G5) even aggravated tumor hypoxia, probably because the catalytic oxidation of GOD consumed oxygen in tumor tissues. We further measured the H_2_O_2_ content in tumor cells after different treatments to evaluate GOD‐mediated catalytic oxidation reactions. As can be seen in Figure 6B, free GOD can only produce a moderate elevation of H_2_O_2_ level (1.4 times higher than control), which may be attributed to the short half‐life of GOD.^[^ ^14a^ ^]^ Comparatively, HCG exhibited a higher H_2_O_2_ accumulation (2.0 times higher than control), which was due to more GOD accumulated in the tumor region with the help of nanoplatform. Dramatically, when combined with HBO, HCG caused a sharp increase in H_2_O_2_ levels within tumor cells, up to 3.1 times that of control group. This result revealed that HBO ameliorated tumor hypoxia, which greatly activated the catalytic oxidation of GOD. Taking into account that we have demonstrated in cellular experiments that HCG can amplify oxidative stress by regulating redox homeostasis (Figures 2 and 3), we further investigated the influence of different treatments on oxidative stress in tumor tissues. For GSH detection, no obvious GSH consumption was observed with HBO, GOD, and HPD treatments, while the HPD‐Cu‐treated group showed moderate GSH reduction owing to Cu^2+^‐mediated redox reaction. In contrast, the GSH content in tumor tissues of HCG‐treated mice was significantly lower than those of other groups (Figure 6C), probably due to the GOD‐triggered glucose deprivation. A similar trend was observed in GSH staining (Figure S20, Supporting Information). Subsequently, ROS levels in tumor tissues following different treatments were detected by flow cytometry using DCFH‐DA as probe. As expected, HCG+HBO combination group induced the highest level of ROS (Figure 6D), consistent with the results of pharmacodynamic experiments (Figure 5B). All these results convincingly verified our anticipation that HBO held strong ability to relieve tumor hypoxia and activate GOD in HCG, amplifying oxidative stress within tumor tissues.

Encouraged by the superior in vitro CSCs inhibition as well as high tumor accumulation penetration, and suppression of HCG, we sought to evaluate the CSCs inhibition effect in orthotopic 4T1 tumor tissues after different treatments. The proportion of CSCs after different treatments was first analyzed by flow cytometry using three representative CSCs characterizing method, CD133^+^, CD44^+^CD24^−^, and side populations (SP). Some consistent conclusions can be drawn from flow cytometric analysis results (Figure 6E–G and Figures S21–S23, Supporting Information). The results of all three CSCs indicators showed that the proportion of CSCs was significantly reduced in the HBO‐treated group, which resulted from the amelioration of tumor hypoxia by HBO.^[^ ^12b^ ^]^ Interestingly, HCG treatment alone also achieved moderate inhibition of CSCs, with the proportions reduced by 35.2% in CD133^+^‐identified CSCs, 40.5% in CD44^+^CD24^−^‐identified CSCs, and 52.5% in SP‐identified CSCs. This phenotype may be because HCG can induce a certain level of oxidative stress, which is lethal to CSCs. To make this convincing, we subsequently tested the ROS levels in CD133^+^ CSCs after different treatments. As depicted in Figure S24 in the Supporting Information, treatment with HCG alone did enhance oxidative stress, which should be responsible for the suppression of CSCs by HCG. When HBO was combined with HCG, the proportion of CSCs in tumor tissues was further diminished. Specifically, the percentage of CSCs identified by CD133^+^, CD44^+^CD24^−^, and SP decreased by 56.3%, 50.8%, and 81.8%, respectively. Besides, CD133 and SOX2 (typical CSCs markers) immunofluorescence staining also yielded similar results that HCG+HBO combination therapy could most efficiently eliminate CSCs (Figure 6H,I). The reason for these results may be that, on the one hand, HBO ameliorates tumor hypoxia and amplifies the oxidative stress of CSCs, and on the other hand, HBO degrades the ECM, which facilitates the accessibility of HCG to CSCs.

### 2.7 Lung Metastasis Evaluation

**Figure 7:**
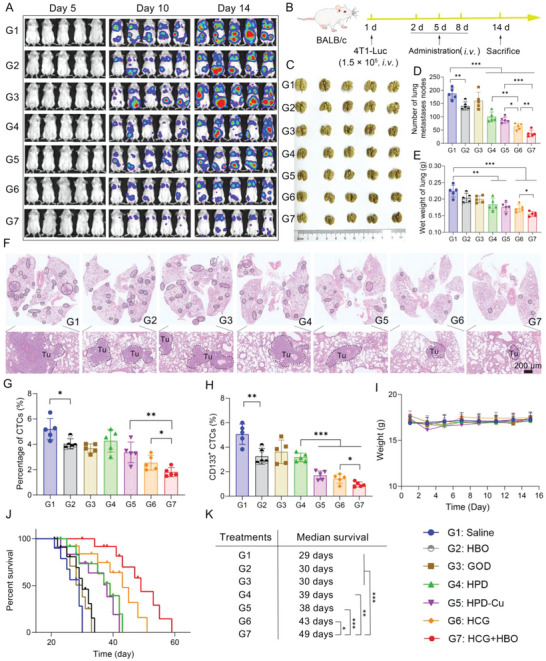
Pulmonary metastasis and survival assessment. A) In vivo bioluminescence images tracking the spreading and growth of intravenously injected 4T1‐Luc cells in the mice after different treatments. B) Schematic illustration of pulmonary metastasis assay. C) Photographs of the gross appearance of the tumor nodules in the lung after different treatments. D) The number of lung nodules in each group. E) Wet weight of lung in different groups. F) Representative H&E staining analysis of the lung metastasis. Scale bars: 200 µm. Percentage of G) circulating tumor cells (CTCs) and H) CD133^+^ CTCs in mice blood after different treatments. I) Body weight surveillance of mice during the treatments. J) Morbidity‐free survival of mice after the indicated treatments, and K) corresponding median survival. Survival curves were analyzed using the log‐rank (Mantel–Cox) test, and the statistical significance of the other experiments was calculated by *t*‐test. *p* values: * *p* < 0.05, ** *p* < 0.01, *** *p* < 0.001.

CSCs are considered as the dominant culprit of cancer metastasis.^[^ ^41^ ^]^ Given that the combination of HCG and HBO enabled potent CSCs elimination both in vitro and in vivo, we further evaluated the effects of various treatments on cancer metastasis. Migration and invasion capabilities are critical in the metastasis cascades. Prior to investigating tumor metastasis in vivo, the degree of antimigration of HCG was assessed by wound healing, tumor migration, and tumor invasion assays. As shown in Figure S25 in the Supporting Information, the control group showed robust wound healing with wound closure rate of 79.3%, indicating that 4T1 cancer cells had inherent metastatic features. The lowest wound closure rate of 27.8% was observed in the HCG‐treated group in normoxic conditions, which implied the most effective inhibition of cell motility. But the capacity of HCG in inhibiting cell motility was significantly reduced in the hypoxic state. When HBO was applied, the inhibitory effect of HCG on cell motility was partially restored. In the similar trend as wound healing assay, HCG exhibited the strongest migration and invasion suppression in normoxic conditions, with rates of 27.9% and 18.7%, respectively. At the same time, hypoxia would attenuate the ability of HCG to inhibit cell migration and invasion, which could also be recovered by HBO. The high antimetastasis effect in vitro drove us to investigate the antimetastatic ability of HCG in vivo. To construct pulmonary metastatic model, mice were intravenously injected with 4T1 cancer cells expressing firefly luciferase (4T1‐Luc). The experimental schedule was presented in **Figure** **7**B. The therapeutic efficacy of different treatments was monitored by bioluminescence imaging. As shown in Figure 7A and Figure S26 in the Supporting Information, the pulmonary bioluminescence signal was intensified to some extent in different groups as time went by. Specifically, the mice treated with saline, HBO, GOD showed strong bioluminescence signals on 14 days, while the bioluminescence signals of HPD, HPD‐Cu, and HCG‐treated mice were significantly weaker than the former three groups. In contrast, the mice treated with HCG plus HBO exhibited the weakest bioluminescence signals, indicating the remarkable inhibition of tumor metastasis. To directly evaluate lung metastasis, lungs were collected from each group on day 14. The gross appearance of whole lungs (Figure 7C) and count of metastatic nodules (Figure 7D) revealed that the formulation of metastatic foci was effectively suppressed in HCG‐treated group (≈64 nodules), compared with control (≈187 nodules), HBO (≈141 nodules), GOD (≈161 nodules), HPD (≈100 nodules), and HPD‐Cu (≈85 nodules)‐treated groups. Encouragingly, only very few lung nodules (≈38) were observed in the HCG+HBO group, suggesting that HCG+HBO combination strategy afforded a strong antimetastatic capability. Meanwhile, the wet weight of lungs in the HCG+HBO‐treated group was significantly lower than all other six groups (Figure 7E), also indicating the least number of metastatic foci. Pathological analysis of the lungs (Figure 7F) showed a similar trend. The mobilization of circulating tumor cells (CTCs) is a necessary step in the metastatic process of solid malignancies.^[^ ^42^ ^]^ And CTCs can be used as an important marker to predict tumor metastasis. For a clearer understanding of the metastasis process, we further analyzed CTCs and CD133^+^ CTCs levels in blood after different treatments by flow cytometry. The results exhibited that the number of CTCs (Figure 7G and Figure S27, Supporting Information) and CD133^+^ CTCs (Figure 7H and Figure S28, Supporting Information) had a significant decrease in mice treated with HCG+HBO, which provided a reasonable explanation for the results of lung metastasis. No significant change in body weight was noticed throughout the lung metastasis treatment period, suggesting the negligible adverse effects of HCG in combination with HBO (Figure 7I). As a result, the mice in HCG+HBO treated group presented the longest survival time among all groups (Figure 7J), with a median survival of 49 days compared to 29 days in the control group (Figure 7K). Collectively, these results highlight the combination of HCG with HBO potently suppresses tumor metastasis and significantly prolongs mice survival by inhibiting intractable CSCs.

## 3 Conclusions

In summary, we rationally designed an acidity‐activatable bioreactor (HCG) through simple self‐assembly approach, which achieved synergistic cancer starvation‐/chemodynamic‐/chemo‐therapy with the support of HBO. The detailed in vitro and in vivo results authenticated that HBO could effectively promote HCG‐triggered tumor starvation, •OH generation, and drug release by alleviating tumor hypoxia. Meanwhile, HBO was proven to possess the ability to degrade dense ECM of aggressive breast cancers, facilitating tumor enrichment, and penetration of HCG. The introduction of HBO increased the tumor suppression rate of HCG by 45%. We demonstrate that the Fenton‐like agent Cu^2+^ in HCG depletes ROS scavenger GSH while GOD‐induced glucose deprivation inhibits NADPH pathway, which collectively reduce the unnecessary waste of ROS and amplify oxidative stress in tumor tissues. The enhanced tumor penetration and the amplified oxidative stress allow HCG to potently eliminate CSCs. As a consequence, HCG+HBO effectively suppresses not only the growth of orthotopic 4T1 breast cancers but more importantly the lethal pulmonary metastasis. Considering the clinical availability of HBO and HES, this work offers an innovative strategy for the synergistic and comprehensive management of cancers, with significant value for future clinical translation.

## Conflict of Interest

The authors declare no conflict of interest.

## Author Contributions

Y.X., Z.Y., and C.X. contributed equally to this work. Z.L. and X.Y. supervised and supported this work. Y.X. and Z.L. conceived this project. Y.X., Z.Y., C.X., Q.D., Q.W., S.L., C.W., and Z.Z. performed the experiments. Y.X. and Z.L. analyzed the data. Y.X. wrote the manuscript. Y.X. and Z.L. contributed to the revision.

## Supporting information

Supporting Information

## Data Availability

The data that support the findings of this study are available from the corresponding author upon reasonable request.

## References

[1] a) W. R. Wilson , M. P. Hay , Nat. Rev. Cancer 2011, 11, 393; 21606941 10.1038/nrc3064

[2] D. Samanta , D. M. Gilkes , P. Chaturvedi , L. Xiang , G. L. Semenza , Proc. Natl. Acad. Sci. U. S. A. 2014, 111, 5429. 10.1073/pnas.1421438111 PMC4273385 25453096

[3] M. R. Horsman , J. Overgaard , J. Radiat. Res. 2016, 57, i90. 26983987 10.1093/jrr/rrw007 PMC4990104

[4] a) W. L. Liu , T. Liu , M. Z. Zou , W. Y. Yu , C. X. Li , Z. Y. He , M. K. Zhang , M. D. Liu , Z. H. Li , J. Feng , X. Z. Zhang , Adv. Mater. 2018, 30, 1802006;

[5] S. Turajlic , C. Swanton , Science 2016, 352, 169. 27124450 10.1126/science.aaf2784

[6] Y. Cheng , H. Cheng , C. Jiang , X. Qiu , K. Wang , W. Huan , A. Yuan , J. Wu , Y. Hu , Nat. Commun. 2015, 6, 8785. 26525216 10.1038/ncomms9785 PMC4659941

[7] J. Yang , W. Li , L. Luo , M. Jiang , C. Zhu , B. Qin , H. Yin , X. Yuan , X. Yin , J. Zhang , Z. Luo , Y. Du , J. You , Biomaterials 2018, 182, 145. 30121013 10.1016/j.biomaterials.2018.08.004

[8] J. Wang , B. Zhang , J. Sun , Y. Wang , H. Wang , Adv. Ther. 2020, 3, 1900083. 10.1002/adtp.201900083 PMC8281934 34277929

[9] a) H. Chen , J. Tian , W. He , Z. Guo , J. Am. Chem. Soc. 2015, 137, 1539; 25574812 10.1021/ja511420n

[10] a) D. Wang , H. Wu , S. Z. F. Phua , G. Yang , W. Qi Lim , L. Gu , C. Qian , H. Wang , Z. Guo , H. Chen , Y. Zhao , Nat. Commun. 2020, 11, 357; 31953423 10.1038/s41467-019-14199-7 PMC6969186

[11] X. Song , J. Xu , C. Liang , Y. Chao , Q. Jin , C. Wang , M. Chen , Z. Liu , Nano Lett. 2018, 18, 6360. 30247918 10.1021/acs.nanolett.8b02720

[12] a) D. M. Brizel , S. Lin , J. L. Johnson , J. Brooks , M. W. Dewhirst , C. A. Piantadosi , Br. J. Cancer 1995, 72, 1120; 7577456 10.1038/bjc.1995.474 PMC2033965

[13] a) S. Yu , Z. Chen , X. Zeng , X. Chen , Z. Gu , Theranostics 2019, 9, 8026; 31754379 10.7150/thno.38261 PMC6857045

[14] a) L. H. Fu , C. Qi , Y. R. Hu , J. Lin , P. Huang , Adv. Mater. 2019, 31, 1808325; 10.1002/adma.201808325 30907460

[15] L. H. Fu , C. Qi , J. Lin , P. Huang , Chem. Soc. Rev. 2018, 47, 6454. 30024579 10.1039/c7cs00891k

[16] T. He , H. Xu , Y. Zhang , S. Yi , R. Cui , S. Xing , C. Wei , J. Lin , P. Huang , Theranostics 2020, 10, 1544. 32042321 10.7150/thno.40439 PMC6993236

[17] C. Qiao , Z. Yang , X. Liu , R. Zhang , Y. Xia , L. Wang , Z. Chen , Q. Jia , R. Wang , Y. Yang , Z. Wang , Nano Lett. 2022, 22, 8250. 36218311 10.1021/acs.nanolett.2c02983

[18] a) F. Duan , W. Jin , T. Zhang , Y. Sun , X. Deng , W. Gao , Adv. Mater. 2023, 2209765, 10.1002/adma.202209765; 36773963

[19] a) B. Ma , S. Wang , F. Liu , S. Zhang , J. Duan , Z. Li , Y. Kong , Y. Sang , H. Liu , W. Bu , L. Li , J. Am. Chem. Soc. 2019, 141, 849; 30541274 10.1021/jacs.8b08714

[20] S. Y. Li , L. H. Liu , H. Z. Jia , W. X. Qiu , L. Rong , H. Cheng , X. Z. Zhang , Chem. Commun. 2014, 50, 11852. 10.1039/c4cc05008h 25145493

[21] L. H. Fu , Y. Wan , C. Qi , J. He , C. Li , C. Yang , H. Xu , J. Lin , P. Huang , Adv. Mater. 2021, 33, 2006892. 10.1002/adma.202006892 33394515

[22] H. Wu , H. Hu , J. Wan , Y. Li , Y. Wu , Y. Tang , C. Xiao , H. Xu , X. Yang , Z. Li , Chem. Eng. J. 2018, 349, 129.

[23] H. H. Peng , D. X. Hong , Y. X. Guan , S. J. Yao , Int. J. Pharm. 2019, 558, 82. 30639222 10.1016/j.ijpharm.2018.12.077

[24] W. Ying , Y. Zhang , W. Gao , X. Cai , G. Wang , X. Wu , L. Chen , Z. Meng , Y. Zheng , B. Hu , X. Lin , ACS Nano 2020, 14, 9662. 32709200 10.1021/acsnano.0c00910

[25] Y. Xiong , Z. Wang , Q. Wang , Q. Deng , J. Chen , J. Wei , X. Yang , X. Yang , Z. Li , Theranostics 2022, 12, 944. 34976222 10.7150/thno.67572 PMC8692913

[26] L. S. Lin , T. Huang , J. Song , X. Y. Ou , Z. Wang , H. Deng , R. Tian , Y. Liu , J. F. Wang , Y. Liu , G. Yu , Z. Zhou , S. Wang , G. Niu , H. H. Yang , X. Chen , J. Am. Chem. Soc. 2019, 141, 9937. 31199131 10.1021/jacs.9b03457

[27] Y. Li , Y. Wu , J. Chen , J. Wan , C. Xiao , J. Guan , X. Song , S. Li , M. Zhang , H. Cui , T. Li , X. Yang , Z. Li , X. Yang , Nano Lett. 2019, 19, 5806. 31331172 10.1021/acs.nanolett.9b02769

[28] S. Liang , X. Xiao , L. Bai , B. Liu , M. Yuan , P. Ma , M. Pang , Z. Cheng , J. Lin , Adv. Mater. 2021, 33, 2100333. 10.1002/adma.202100333 33792083

[29] Y. Ma , Z. Su , L. Zhou , L. He , Z. Hou , J. Zou , Y. Cai , D. Chang , J. Xie , C. Zhu , W. Fan , X. Chen , S. Ju , Adv. Mater. 2022, 34, 2107560. 10.1002/adma.202107560 34902181

[30] a) N. Hay , Nat. Rev. Cancer 2016, 16, 635; 27634447 10.1038/nrc.2016.77 PMC5516800

[31] X. Guo , F. Liu , J. Deng , P. Dai , Y. Qin , Z. Li , B. Wang , A. Fan , Z. Wang , Y. Zhao , ACS Nano 2020, 14, 14715. 33156626 10.1021/acsnano.0c00764

[32] X. Liu , N. Ye , S. Liu , J. Guan , Q. Deng , Z. Zhang , C. Xiao , Z. Y. Ding , B. X. Zhang , X. P. Chen , Z. Li , X. Yang , Adv. Sci. 2021, 8, 2100233. 10.1002/advs.202100233 PMC8336507 34085419

[33] M. Hockel , P. Vaupel , J. Natl. Cancer Inst. 2001, 93, 266. 11181773 10.1093/jnci/93.4.266

[34] Y. Pan , L. Liu , L. Rao , X. Chen , Matter 2022, 5, 1367.

[35] Z. Q. Zuo , K. G. Chen , X. Y. Yu , G. Zhao , S. Shen , Z. T. Cao , Y. L. Luo , Y. C. Wang , J. Wang , Biomaterials 2016, 82, 48. 26751819 10.1016/j.biomaterials.2015.12.014

[36] a) M. Luo , L. Shang , M. D. Brooks , E. Jiagge , Y. Zhu , J. M. Buschhaus , S. Conley , M. A. Fath , A. Davis , E. Gheordunescu , Y. Wang , R. Harouaka , A. Lozier , D. Triner , S. McDermott , S. D. Merajver , G. D. Luker , D. R. Spitz , M. S. Wicha , Cell Metab. 2018, 28, 69; 29972798 10.1016/j.cmet.2018.06.006 PMC6037414

[37] T. Lang , Y. Liu , Z. Zheng , W. Ran , Y. Zhai , Q. Yin , P. Zhang , Y. Li , Adv. Mater. 2019, 31, 1806202. 10.1002/adma.201806202 30516854

[38] Y. Pan , X. Ma , C. Liu , J. Xing , S. Zhou , B. Parshad , T. Schwerdtle , W. Li , A. Wu , R. Haag , ACS Nano 2021, 15, 15069. 34420298 10.1021/acsnano.1c05452

[39] a) J. Liu , Y. Tan , H. Zhang , Y. Zhang , P. Xu , J. Chen , Y. C. Poh , K. Tang , N. Wang , B. Huang , Nat. Mater. 2012, 11, 734; 22751180 10.1038/nmat3361 PMC3405191

[40] Y. Xiong , W. Wang , Q. Deng , Z. Zhang , Q. Wang , Z. Yong , C. Sun , X. Yang , Z. Li , Nano Today 2023, 49, 101767.

[41] a) T. Oskarsson , E. Batlle , J. Massague , Cell Stem Cell 2014, 14, 306; 24607405 10.1016/j.stem.2014.02.002 PMC3998185

[42] V. Plaks , C. D. Koopman , Z. Werb , Science 2013, 341, 1186. 24031008 10.1126/science.1235226 PMC3842225

