# Supplementary material for: Hyperbaric Oxygen Activates Enzyme‐Driven Cascade Reactions for Cooperative Cancer Therapy and Cancer Stem Cells Elimination
Source: Adv Sci (Weinh). 2023 Apr 28;10(21):2301278. doi: 10.1002/advs.202301278 (PMC10375084; doi:10.1002/advs.202301278)
Supplement: Supplementary file 1 — Supporting Information [file ADVS-10-2301278-s001.pdf]

ADVANCED SCIENCE Open Access

Supporting Information

for *Adv. Sci.*, DOI 10.1002/advs.202301278

Hyperbaric Oxygen Activates Enzyme-Driven Cascade Reactions for Cooperative Cancer Therapy and Cancer Stem Cells Elimination

Yuxuan Xiong, Zhengtao Yong, Chen Xu, Qingyuan Deng, Qiang Wang, Shiyou Li, Chong Wang, Zhijie Zhang, Xiangliang Yang and Zifu Li**

Supporting information for

Hyperbaric Oxygen Activates Enzyme-driven Cascade Reactions for Cooperative Cancer Therapy and Cancer Stem Cells Elimination

Yuxuan Xiong[a#], Zhengtao Yong[a#], Chen Xu[a#], Qingyuan Deng[a], Qiang Wang[a], Shiyou Li[a], Chong Wang[a], Zhijie Zhang[a], Xiangliang Yang[a,b,c,d,e,f]*, Zifu Li[a,b,c,d,e]*

[a] National Engineering Research Center for Nanomedicine, College of Life Science and Technology, Huazhong University of Science and Technology, Wuhan, 430074, P. R. China

[b] Key Laboratory of Molecular Biophysics of Ministry of Education, College of Life Science and Technology, Huazhong University of Science and Technology, Wuhan, 430074, P. R. China

[c] Hubei Key Laboratory of Bioinorganic Chemistry and Materia Medical, Huazhong University of Science and Technology, Wuhan, 430074, P. R. China

[d] Hubei Engineering Research Center for Biomaterials and Medical Protective Materials, Huazhong University of Science and Technology, Wuhan, 430074, P. R. China

[e] Hubei Bioinformatics and Molecular Imaging Key Laboratory, College of Life Science and Technology, Huazhong University of Science and Technology, Wuhan, 430074, P. R. China

[f] GBA Research Innovation Institute for Nanotechnology, Guangdong, 510530, P. R. China

[#] These authors contributed equally.

* Correspondence and requests for materials should be addressed to ZFL (email: zifuli@hust.edu.cn) or XLY (email: yangxl@hust.edu.cn).

Experimental Section

Materials. Hydroxyethyl starch (HES, Mw: 130 kDa, hydroxyethyl molar substitution degree: 0.4) was a gift from Wuhan HUST life Science & Technology Ltd. (Wuhan, China). 4-Nitrophenyl chloroformate and hydrazine monohydrate ($N_2H_4.H_2O$) were purchased from Energy Chemical. Glucose oxidase (GOD, > 180 U/mg), 5,5-Dimethyl-1-pyrroline N-oxide (DMPO), methylene blue (MB), glutathione (GSH), 5,5'-Dithiobis(2-nitrobenzoic acid (DTNB) and copper (II) chloride dihydrate ($CuCl_2.2H_2O$) were bought from Aladdin Reagent (Shanghai) Co., Ltd. Doxorubicin (99%) was obtained from Beijing Huafenglianbo Technology Co., Ltd. (Beijing, China). Sodium hydroxide (NaOH), dimethyl sulfoxide (DMSO), N,N-Dimethylformamide (DMF), isopropyl alcohol (IPA), petroleum ether (PE) and hydrogen peroxide (H_2O_2, 30%) were purchased from SinopharmChemical Reagent Co., Ltd, (Shanghai, China). Annexin V-APC/7-AAD apoptosis kit was purchased from Multisciences (LianKe) Biotech, Co., Ltd. Crystal violet, 2, 7-Dichlorodi-hydrofluorescein diacetate (DCFH-DA) and Calcein-AM/PI double stain kit were bought from Yeasen Biotechnology (Shanghai) Co., Ltd. ATP assay kit, GSH assay kit, $NADP^+$/NADPH assay kit, Hoechst 33342, BCECF-AM, Lyso-Tracker green and H_2O_2 assay kit were purchased from Beyotime Institute of Biotechnology (Shanghai, China). Flow cytometry antibodies included APC anti-mouse CD133 (141208), FITC anti-mouse CD24 (101806), PE/Cyanine7 anti-mouse/human CD44 (103030), PE/Cyanine7 anti-mouse CD31 (102417), and PE anti-mouse CD326 (Ep-CAM) (118206), were purchased from Biolegend. Ultrapure water (Millipore Milli-Q grade, 18.2 MΩ) was used for all experiments. All chemicals were of analytical grade and used without further purification.

Cell lines and animals. The murine breast cancer cell line 4T1 and murine embryonic cell line NIH-3T3 were acquired from BeNa Culture Collection. Cells were cultured according to the method recommended in the instructions. Female BALB/C mice (SPF, 16-20 g) were obtained from Beijing Vital River Laboratory Animal Technology Co. Ltd. All animal procedures were performed in strict accordance with the internationally accepted principles and guidelines for the Care and Use of Laboratory Animals of Huazhong

University of Science and Technology and the experiment protocols were approved by the Institutional Animal Ethical Committee of Huazhong University of Science and Technology. The mice were placed in a specific pathogen-free environment and have free access to food and water.

Synthesis of pH-responsive prodrug HES-Hyd-DOX.

(1) Synthesis of intermediates HES-NPC. In brief, 1 g HES was dispersed in 24 mL of ultrapure water and stirred under ice-water bath. Then, 1 mL NaOH solution (2M) was added to the reaction system. After vigorously stirring for 1 min, 400 mg 4-Nitrophenyl chloroformate (NPC) dissolved in 10 mL dichloromethane was added dropwise to the mixture. After that, the reaction system was transferred to room temperature and reacted for 1 h under stirring. The crude product HES-NPC was obtained by repeated precipitation with large amount of isopropyl alcohol and petroleum ether. The precipitates were redissolved in DMSO and dialyzed with ultrapure water for 3 days. After freeze-drying, the obtained product was stored at 4°C for further use. The yield of HES-NPC was 75.7%.

(2) **Synthesis of intermediates HES-Hyd.** HES-NPC (0.8 g) was firstly dispersed in 10 mL DMSO. Then, 0.8 mL hydrazine monohydrate was added under vigorous stirring. After 5 h reaction at room temperature, the crude product of HES-Hyd was collected by repeated precipitation with abundant isopropyl alcohol and petroleum ether. HES-Hyd was further purified by dialysis for 3 days. After lyophilization, the resulting product was stored at 4°C for further use. The yield of HES-Hyd was 89.4%.

(3) **Synthesis of HES-Hyd-DOX.** 0.6 g HES-Hyd was dispersed in 10 mL DMF. Then 120 μL triethylamine and 60 μL acetic acid were added to the reaction system sequentially. Next, 0.1 g DOX dissolved in 2 mL DMF (containing 20 μL triethylamine) was added to the mixture. The reaction was kept under nitrogen atmosphere and stirred vigorously at room temperature for 48 h. The crude product of HES-Hyd-DOX was obtained by repeated precipitation with abundant isopropyl alcohol and petroleum ether. The HES-Hyd-DOX was further purified by dialysis for 3 days, followed by lyophilization. The yield of HES-Hyd-DOX was calculated to be 72.6%.

Preparation of HCG. For the preparation of HCG, 2.0 mg GOD was dissolved in 8 mL ultrapure water with a final concentration of 0.25 mg/mL. Then 100 mg HES-Hyd-DOX

containing 7.7 mg DOX was added to the above solution under sonication conditions. After that, a $CuCl_2$ aqueous solution (50 mM, 2 mL) was injected into the mixture using micro-infusion pump at a speed of 1 mL/h under magnetic stirring. The solution gradually changes from orange-red to purple after the addition of $CuCl_2$ aqueous solution. After stirring for an additional 10 h in the dark, HCG were further purified by dialysis (MWCO: 300 kDa) and then lyophilized for future use. HPD-Cu was obtained using the same method without addition GOD.

Material characterization. The morphology and shape of the prepared NPs was recorded using transmission electron microscopy (TEM, HT7700 HITACHI Co., Japan) with a field emission gun operating at 120 kV. The hydrodynamic size and zeta potential was detected via a dynamic light scattering (DLS) device (Malvern Zetasizer Nano ZS90, UK). The FTIR spectra were acquired on a Fourier transform infrared spectroscopy (FTIR, VERTEX 70, Bruker Co., Germany). ^{1}H NMR spectra were measured on a nuclear magnetic resonance spectrometer (AscendTM 600 MHz, Bruker) using deuterated dimethyl sulfoxide (DMSO-d6) as the solvent. The absorption spectra were obtained from a UV-Vis spectrophotometer (Lambda 35, PerkinElmer Instruments Co., Ltd., Shanghai, China). High-resolution transmission electron microscopy (HRTEM) images were obtained with a FEI Tecnai F20 (FEI, Netherlands) at a voltage of 200 kV. The X-ray photoelectron spectra (XPS) were taken on an AXIS-ULTRA DLD-600W electron spectrometer. ESR measurements were carried out using a Bruker electron spin resonance (ESR) spectrometer (EMXmicro-6/1/P/L) at ambient temperature. Inductively coupled plasma optical emission spectrometer (ICP-OES, PerkinElmer Ltd., Co., USA) was utilized to measure the concentration of Cu.

Stability assay of HCG. HCG were dispersed in different media, including ultrapure water, PBS (0.01 M), saline and RPMI-1640 (10% FBS). During incubation at 37 °C, the particle size of HCG was monitored by DLS.

GSH consumption capability of HCG. DTNB was employed to evaluate the GSH consumption behavior of HCG. Briefly, HCG ([Cu] = 0.5 mM) were mixed with GSH (1 mM) in PBS solution at room temperature. At predetermined time periods, 100 μL of the mixed solution was pipetted into 896 μL of PBS solution, followed by the addition of 4 μL of

DTNB stock solution (10 mg/mL). The absorbance spectrum (300-600 nm) of the mixture was measured on a UV-Vis spectrophotometer. DTNB solution was used as a control group under the same conditions.

Release of DOX from HCG. The drug release assay was conducted by dialysis method. 1 mL HCG solution ([DOX] = 320 μg/mL) was packaged into a dialysis tube (molecular weight cutoff = 3500). The tubes were then immersed within 30 mL buffer solutions (pH 7.4, pH 6.5, pH 5.0, containing 0.5% v/v Tween-80) and gently shaken at 100 rpm in a shaker at 37 °C. At predetermined time intervals, 200 μL external buffer solution was taken out and replaced with fresh buffer solution. The concentration of DOX released was analyzed by a multi-mode microplate reader with excitation wavelength at 483 nm and emission wavelength at 556 nm. Each release time point sets three repetitions.

Catalytic capacity of HCG under different pH conditions. The generation of H_2O_2 was used to evaluate the catalytic capacity of HCG. To begin with, HCG were incubated in different buffer solutions (pH 7.4, pH 6.5, pH 5.0) for 24 h. The glucose (5 mM) was then added to the above HCG solutions. The H_2O_2 concentration was measured using a commercial assay kit (Beyotime).

Chemodynamic activity of HCG. Hydroxyl radicals (·OH) were detected by methylene blue (MB). Specifically, HCG ([Cu] = 1 mM) was mixed with 1 mM GSH in 890 μL buffer solution (pH 6.5). The mixture was then shaken in the dark for 2 h at 37 °C. After that, MB (10 μL, 1 mg/mL) and glucose (100 μL, 50 mM) were added into the mixed solution followed by incubation for 5 h at 37 °C. The amount of ·OH production was reflected by the decrease in absorbance at 660 nm.

The generation of ·OH was also verified by ESR spectroscopy using DMPO as radical trapper agent. With 10 μL DMPO (1 M) in a dark Eppendorf tube, the following samples were added: (1) H_2O, (2) HCG, (3) HCG + Glucose, (4) HCG + Glucose +GSH ([Cu] = 1mM, [Glucose] = 5 mM, [GSH] = 1 mM, total volume of each sample is 90 μL). Immediately thereafter, the solution was transferred to a quartz capillary and the ESR signal was detected at room temperature.

***In vitro* antitumor therapy.** For cytotoxicity evaluation, 4T1 cancer cells were seeded in

96-well plates with an initial seeding intensity of 5×10^3 cells per well and incubated in 5% CO_2 at 37 °C for 24 h. For *in vitro* therapy, 4T1 cancer cells were incubated with HPD, HPD-Cu, and HCG at a range of concentrations at 37 °C under normoxic for 24 h. The relative cell viability was determined by standard MTT assay.

For the *in vitro* cytotoxicity under different glucose levels, 4T1 cancer cells were incubated with HCG under various glucose concentration (0, 1, 2, 5 mM) for 24 h. Standard MTT assay was used to determine the cell viability.

Live and dead cell observation by CLSM. 4T1 cancer cells were incubated in confocal dishes (2×10^5 cells per well) until adherence. Subsequently, the culture medium was removed. The cells were incubated with fresh medium containing PBS, HPD, HPD-Cu, GOD, HCG w/o glucose and HCGs w/ glucose ([DOX] = 1 μg/mL). After co-culture for 24 h, the cells were stained with Calcein AM and 7-AAD according to the product description for 0.5 h and then observed using CLSM.

Apoptosis and necrosis assay *in vitro*. 4T1 cancer cells were seeded in 6-well plates and incubated until adherence. After which, previous medium was replaced by fresh medium that contained PBS, HPD, HPD-Cu, GOD, HCG w/o glucose and HCG w/ glucose ([DOX] = 1 μg/mL) for 24 h co-culture. Thereafter, all treated cells were harvested and quantified by apoptosis with an Annexin V-APC/7-AAD apoptosis detection kit using flow cytometer.

Intracellular ROS detection. DCFH-DA was employed to detect the intracellular ROS within the cells. Briefly, 4T1 cancer cells were seeded in confocal dishes at a density of 2×10^5 cells/well. After 24 h incubation, the cells were stained with DCFH-DA (10 μM) for 30 minutes. Following that, previous medium was replaced by fresh one containing PBS, HPD, HPD-Cu, GOD, HCG w/o glucose and HCG w/glucose ([DOX] = 1 μg/mL) in the dark. After culturing for 6 h, all treated cells were gently washed with PBS and then observed using CLSM. Besides, flow cytometer was also used to quantify the ROS levels after different treatments.

Intracellular ATP assay. 4T1 cancer cells were inoculated into the 6-well plate and cultured until adherence. After different treatments, the cellular ATP content was determined with luminescent ATP assay kit according to the manufacturer's instructions.

The measurements were normalized by cell number and independently repeated three times.

Intracellular H_2O_2 detection. 4T1 cancer cells were maintained in 6-well plates with 2×10^5 cells per well for 24 h. The cells were then treated with PBS, HPD, HPD-Cu, GOD, HCG w/o glucose and HCGs w/ glucose ([DOX] = 1 μg/mL), respectively. After 12 h of incubation, the cells were collected and counted, and then tested for intracellular H_2O_2 content based on the assay kit protocol.

Intracellular redox state analysis. The $NADP^+$/NADPH ratio in cells was determined using a commercially available $NADP^+$/NADPH assay kit. In brief, 4T1 cancer cells were seeded into 6-well plates at a density of 2×10^5 cells per well and incubated for 24 h. Then different drugs were added and incubated for 6 h. The intracellular $NADP^+$/NADPH ratio was measured according to the manufacturer's instructions. For intracellular GSH assay, the 4T1 cells with various treatments were collected according to the above experimental procedure. Afterwards, the intracellular concentration of GSH was measured using a GSH Assay Kit. All the results were normalized by cell number and independently repeated three times.

Cellular endosomal/lysosomal escape assay. In a confocal dish, 1 $\times10^5$ cells per well were seeded and allowed to adhere for 24 h. After incubation with HCG ([DOX] = 1 μg/mL) for 1, 4 and 8 h, lysosomal tracking green (50 nM) was added and stained for 30 min. The cells were then washed with PBS and observed with CLSM.

HBO-assisted cell viability assay. The cytotoxicity of HCGs under hypoxic and HBO conditions was evaluated. Specifically, 4T1 cells were seeded into 96-well plates at a density of 5×10^3 cells and cultured for 24 h. Then, the cells were treated with serial dilutions of HCG and further incubated under hypoxic condition for 24 h. For HBO-treated group, the cells were treated with HBO (2.5 ATA) for 1.5 h after 12 h of hypoxic cultivation. Standard MTT assay and flow cytometric analysis were performed to assess cytotoxicity.

In addition, intracellular biomarkers such as H_2O_2 concentration, ATP level and ROS content in cells treated with HCG under hypoxic as well as hypoxic + HBO conditions were measured by specific assay kits mentioned above.

Intracellular pH detection. BCECF-AM, a pH fluorescent probe, was utilized to detect pH

changes after different treatments. The 4T1 cancer cells seeded on confocal dishes were treated with HCG ([DOX] = 0.5 μg/mL) under hypoxia or hypoxia + HBO (2.5 ATA, 1.5 h) condition for 12 h. After incubation, the cells were gently washed with PBS and stained with BCECF-AM (2 μM) for 30 min. The fluorescence of BCECF was observed by CLSM.

Cell viability assay of 2D cultured CSCs. 2D cultured cancer stem cells (CSCs) were sorted from 4T1 cancer cells and cultured according to the guidance of previous work.[1] For mammosphere culture, 4T1 cancer cells (1000 cells/mL) were cultured in suspension with serum-free DMEM/F12 (Hyclone), complemented with B27 (Gibco), 20 ng/mL bFGF (ProSpec), 20 ng/mL hEGF (ProSpec), 0.4% low endotoxin bovine serum albumin (Sigma-Aldrich) and 4 μg/mL of insulin (Sigma-Aldrich). The mammospheres were collected by gentle centrifugation after 5 d of incubation, followed by digestion (0.05% trypsin-EDTA) into single cells for subsequent cytotoxicity testing.

The Calcein AM/7-AAD was employed to visualize the cytotoxicity of various treatments on 2D cultured CSCs. Briefly, 2D cultured CSCs were inoculated into an ultralow attachment 6 well plates (Corning) at a density of 2×10^4 cells per well. After pre-incubation for 3 d, the cells were treated with PBS, HPD, HPD-Cu, GOD, HCG for 24 h. The cells were then co-stained with Calcein AM/7-AAD and visualized by CLSM.

For cell viability assay, 2D cultured CSCs were seeded on ultra-low attachment 96 well plates (Corning) at 5×10^3 cells per well and further cultured for 3 d. Afterwards, the cells were treated with different concentrations of HCGs under hypoxia or hypoxia + HBO (2.5 ATA, 1.5 h) condition. After 24 h incubation, 100 μL of Cell Counting-Lite 3D solution was added to each well and incubated for another 30 min. The cell viability was measured by molecular devices flexstation3 (FlexS3).

3D cultured CSCs assay. The CSCs were screened in soft 3D fibrin gels (90 Pa) as reported in previous work.[2] 4T1 cancer cells were seeded in 12-well plates and incubated for 24 h. Then the cells were subjected to different treatments, including PBS, HPD, HPD-Cu, GOD, HCG (Normoxia), HCG (Hypoxia), HCG (Hypoxia + HBO) ([DOX] = 0.5 μg/mL). After co-culture for 6 h, the cells were digested with 0.25% trypsin into a single cell suspension at a concentration of 4×10^4 cells/mL. Immediately afterwards, 2 mg/mL fibrinogen (diluted with T7 buffer containing 50 mM Tris and 150 mM NaCl, pH 7.4) was

mixed with the same volume of cell suspension to obtain a fibrinogen/cell mixture. Then, 50 μL fibrinogen/cell mixture (2×10^4 cells/mL) was inoculated in 96-well plates separately, and 1 μL thrombin (0.1 U/μL) was pre-spiked in each well and mixed well. The plates were incubated in a cell incubator for 30 min until gel formation, and then 200 μL of 1640 medium containing 10% fetal bovine serum was added. The colonies were viewed with a microscope and the colony size and number were recorded in a 7-day observation period.

Hemolysis assay. 1.5 mL fresh mouse blood was gathered in an Eppendorf tube containing anticoagulant. The blood samples were then diluted three times with PBS and centrifuged at 3000 rpm for 10 min to acquire RBCs. The RBCs were further rinsed several times followed by 10-fold dilution of purified RBCs with PBS for subsequent use. Different concentrations of HCG solutions were mixed with RBCs and incubated at 37°C for 4 h. The samples were then centrifuged at 12,500 rpm for 10 min to collect the supernatant, and the absorbance at 540 nm was measured to identify the amount of hemoglobin released. Considering that HCG are difficult to be centrifuged, HCG solution without RBCs was used as blank control. The RBCs treated with PBS and 1% TritonX-100 were established as negative and positive controls, respectively. The hemolysis rate was calculated as follows:

$$\text{Hemolysis rate (\%)} = \frac{A_{\text{sample}} - A_{\text{negative}} - A_{\text{blank}}}{A_{\text{positive}} - A_{\text{negative}}} \times 100\%$$

A_{sample}, $A_{positive}$, $A_{negative}$, and A_{blank} stand for the absorbance at 540 nm of the HCGs treated group, TritonX-100 treated group, PBS treated group and pure HCGs (w/o RBCs) treated group.

Pharmacokinetics and biodistribution analysis. Three 4T1 tumor-bearing mice were intravenously injected with 200 μL of HCG solution ([Cu] = 2.25 mg/kg). Subsequently, 20 μL blood was collected from tail vein at desired time points and digested overnight. After dilution, the concentration of Cu in the samples was determined by ICP-OES.

For biodistribution study, the 4T1 tumor-bearing mice (n=3) were intravenously injected with HCGs ([Cu] = 2.25 mg/kg) and sacrificed after 6 h, 12 h, 24 h, 48 h, 72 h, 168 h post injection. The main organs (heart, liver, spleen, lung, kidney) and tumors were dissected, and digested with HNO_3 and $HClO_4$ (v/v=4:1) at 300 ℃ until to form a clear solution. The

biodistribution of HCG was calculated and plotted in unit of % ID/g according to the following equation (1):

$$\%\mathrm{ID/g} = \frac{\mathrm{Cu_{tissue\ lysate}} - \mathrm{Cu_{blank}}}{\mathrm{Cu_{injected}} \times \mathrm{W_{tissue}}} \times 100\% \quad (1)$$

The impact of HBO on biodistribution was further investigated. 4T1 tumor-bearing mice (n = 3) were pretreated daily with HBO (1.5 h, 2.5 ATA) and then intravenously injected with HCG ([Cu] = 2.25 mg/kg). To determine the accumulation of HCGs in tumors and various organs, mice were sacrificed 12 h after injection, and major organs and tumors were harvested, weighted and digested. The mice without HBO treatment were used as control. The concentration of Cu in main organs and tumors was measured by ICP-OES.

Pharmacodynamics evaluation. Orthotopic 4T1 tumor model was established to conduct anti-tumor experiments. Specifically, the skin around the right fourth abdomen of mice was surgically incised, and then single 4T1 cell suspension (100 μL, 5 × 10^6 cells/mL) was injected into the abdominal adipose pad. When the tumor volume reached around 60 mm^3, the mice were randomly divided into 7 groups (n = 6) and subjected with different treatments: (G1) Saline, (G2) HBO, (G3) GOD, (G4) HPD, (G5) HPD-Cu, (G6) HCG, (G7) HBO + HCG. The injected operations (200 μL, [DOX] = 3.12 mg/kg, [GOD] = 0.50 mg/kg) were performed at 2, 5, 8, 11 days by tail vein injection. The mice in G2 and G7 received HBO treatments (2.5 ATA, 1.5 h) on day 1 and 12 h after administrations. Tumor volume (V) was determined by measuring tumor length (L) and width (W) and was calculated as $V = L \times W^2/2$. The tumor volume of mice was measured every other day. At the end of the treatments, the mice were euthanized, the tumors were collected, photographed and weighed.

Identification of CSCs in tumor tissues. After various treatments (at day 12), mice were sacrificed (n=4), the tumors were collected, and CSCs in tumor tissues were detected. Firstly, the tumors were cut as small as possible, supplemented with collagenase IV (Biosharp) and DNase I (Biosharp), and then incubated at 37°C for 1 h. Then, the digested tumor tissues were filtered through a 70 μm cell strainer to obtain single cell suspensions. After that, the cells were washed twice with PBS, resuspended in 300 μL PBS, and stained with the corresponding antibodies from Biolegend. CSCs were identified

by $CD44^+CD24^-EpCAM^+$, $CD133^+$ cell staining and side population cell analysis. Specifically, for $CD44^+CD24^-EpCAM^+$ or $CD133^+$ assay, CSCs were screened with PC7 anti-mouse/human CD44 (Biolegend, 103030), FITC anti-mouse CD24 (Biolegend, 101806) and PE anti-mouse EpCAM (Biolegend, 118206) or APC anti-mouse CD133 (Biolegend, 141208) staining. For side population analysis, cell was incubated with 5 μg/mL Hoechst 33342 alone or in combination with 400 μM verapamil at 37°C for 90 min in the dark. After staining, the cell samples were analyzed by a 4-laser, 13-color BD CytoFlex cytometer.

Besides, DCFH-DA was also used to detect the ROS in tumor tissues according to the protocol of the cell experiment. ROS in CSCs was determined by CD133 and DCFH-DA staining.

Apoptosis and necrosis assay *in vivo*. The single cell suspensions of different tumor tissues were obtained from the above experiments. The cell samples were co-stained with Annexin V-APC and 7-AAD for 30 min in the dark. After rinsing with PBS, cell apoptosis was detected by flow cytometry.

GSH and H_2O_2 detection of tumor tissues. The tumor tissues were firstly cut into small pieces, weighed and homogenized (n=4). The GSH and H_2O_2 levels in tumor cells were measured according to the instructions of the corresponding assay kits (Beyotime).

Immunofluorescence staining and imaging. To characterize tumor hypoxia, tumor sections were immunofluorescent stained with an anti-HIF-1α fluorescently labeled antibody (PTG: 20960-1-AP). CD133 (Abclone: A0219) and Sox2 (Abcam: ab92494) immunofluorescence staining was used to visualize CSCs. To characterize the effect of HBO on ECM, MASSON staining, fibronectin (Abcam: ab199056) and collagenⅠ(Biossci: PA1026) immunofluorescence staining were conducted. The immunofluorescence of tumor tissues was observed by CLSM.

Biosafety evaluation. During the treatments, the body weight of mice was recorded every two days. At day 12, the mice were sacrificed and major organs were obtained for hematoxylin and eosin (H&E) staining. Meanwhile, the blood samples were collected for serum biochemical analysis.

Pulmonary metastasis assay. To establish pulmonary metastasis model, luc-4T1 cells (1.5×10^5) were administered via the tail vein into BALB/c mice. The intravenous tail injection (200 μL, [DOX] = 3.12 mg/kg, [GOD] = 0.50 mg/kg) was conducted at 2, 5, 8 d, and HBO (2.5 ATA, 1.5 h) was performed 12 h after administrations. For the bioluminescence observation, the mice were injected intraperitoneally with D-luciferin and then visualized by a Xenogen IVIS imaging system. On day 14, lungs were collected and fixed in Bouin's solution. The pulmonary nodules were counted and recorded.

Then, the fixed lungs were cut and stained with hematoxylin-eosin for immunohistochemical analysis. In addition, flow cytometry was used to detect the number of circulating tumor cells (CTCs) and $CD133^+$ CTCs in the blood.

Statistical analysis. The statistical analysis was performed using GraphPad Prism (version 8.3.0). All results are presented as means ± standard deviation (S.D.). Significances between different groups were calculated using the unpaired two-tailed student's t test. Survival curves were analyzed using the log-rank (Mantel-Cox) test. Statistical values are indicated according to the following scale: p values: * $p<0.05$, ** $p<0.01$, *** $p < 0.001$, ns stands for not significant.

Supplementary figures

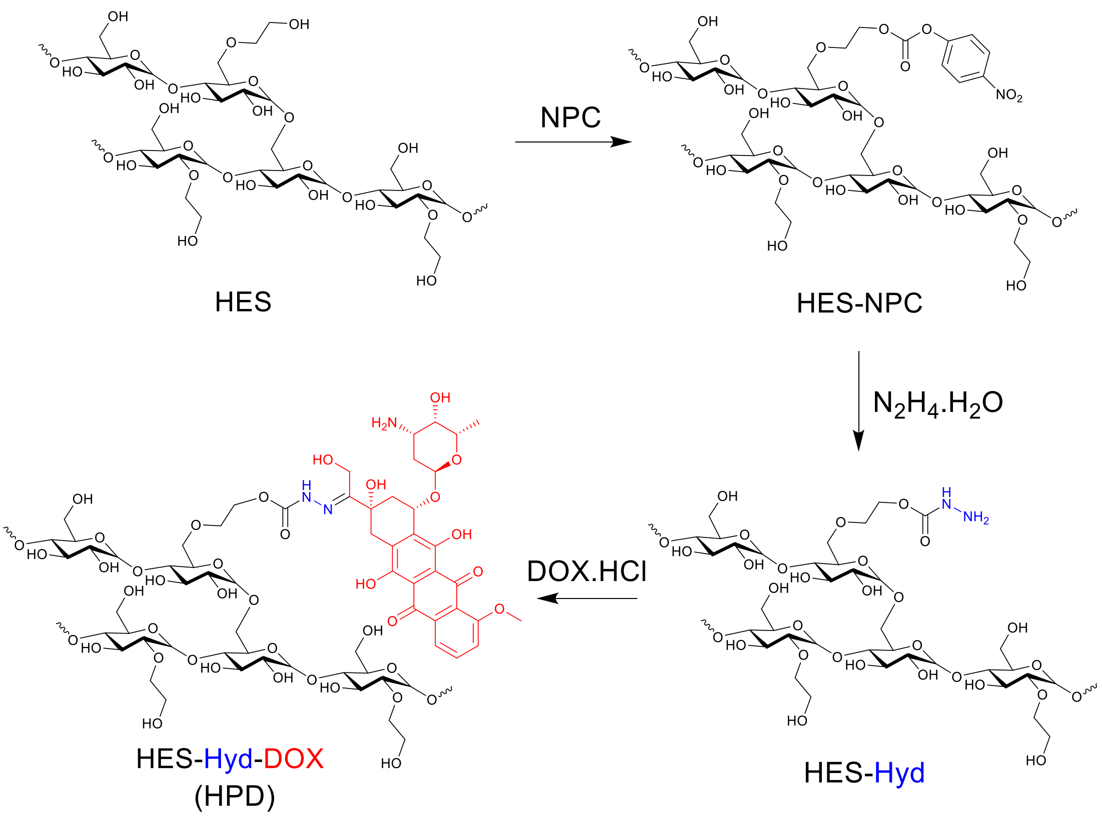

Figure S1. Synthetic route of HES-based pH-responsive prodrug HPD.

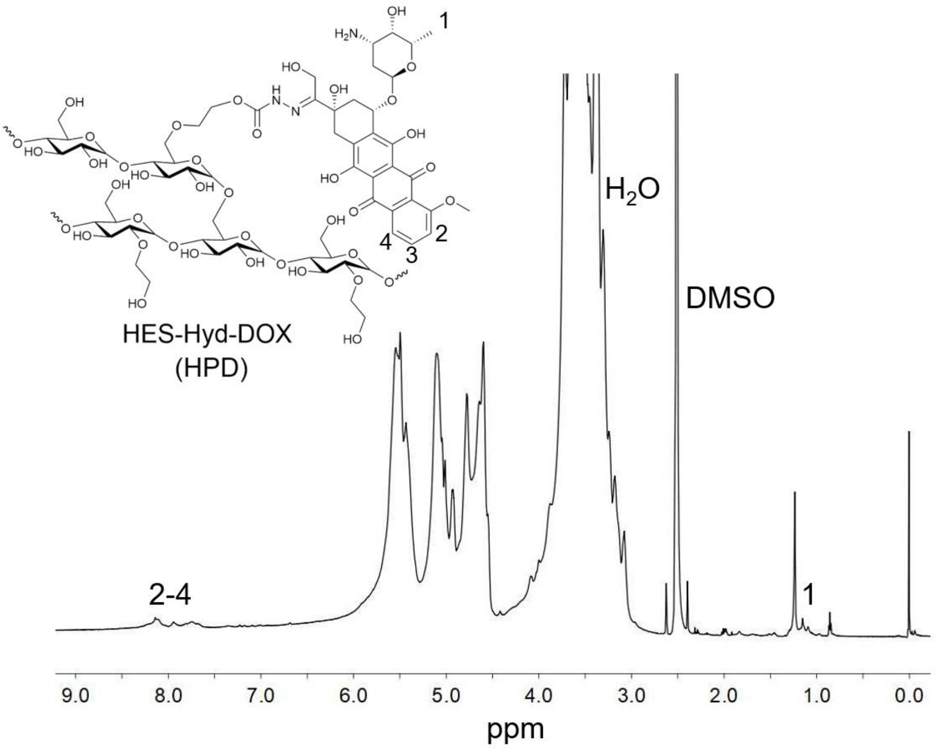

Figure S2. ^1H NMR spectra of HES-Hyd-DOX. The peaks at 1.2, 7.6, 7.9, and 8.1 ppm

correspond to the protons of the methyl group (1) and aromatic ring (2-4) of DOX.[3]

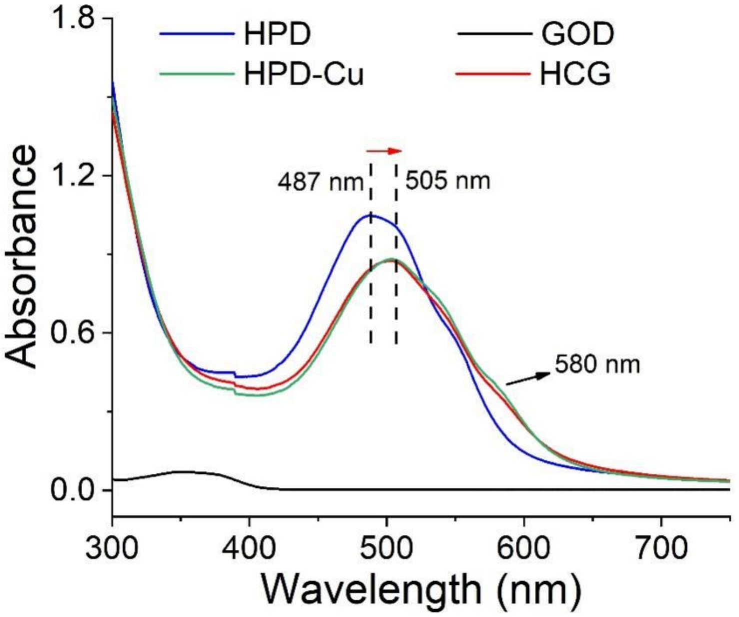

Figure S3. The UV-Vis absorption spectra of HPD, GOD, HPD-Cu, and HCG.

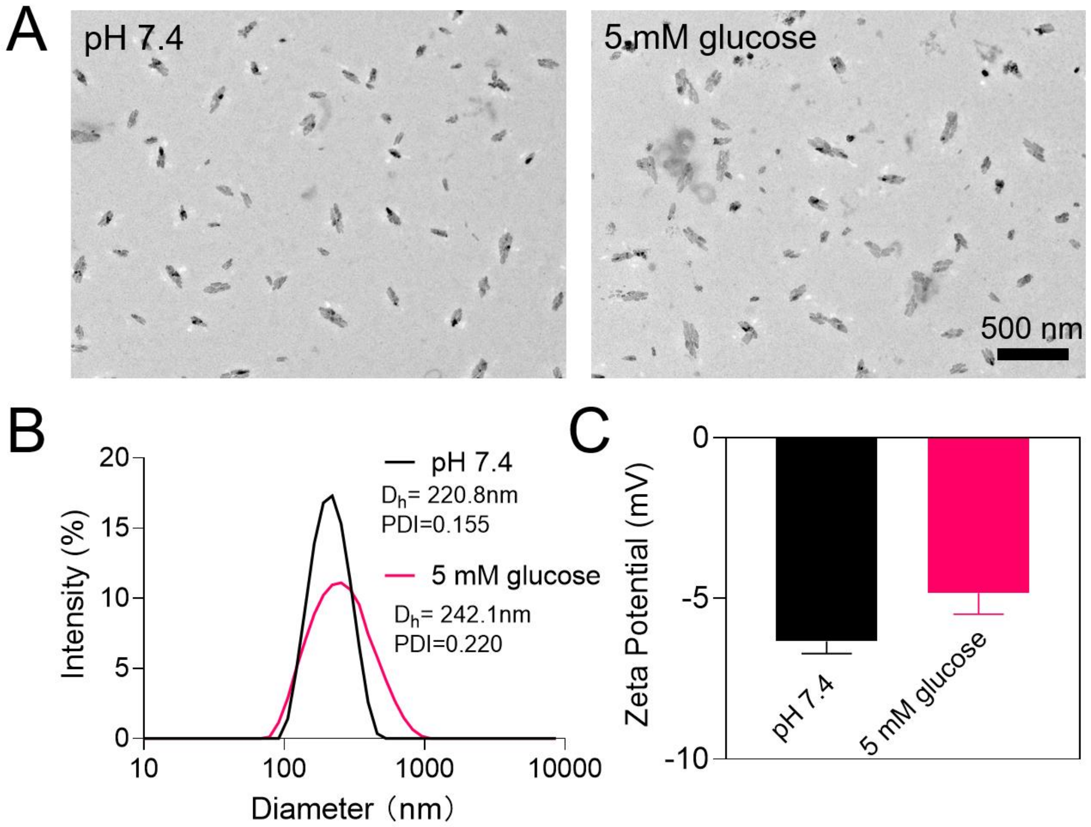

Figure S4. (A) TEM images, (B) DLS size distribution and (C) zeta potentials of HCG in different media. Scale bars: 500 nm.

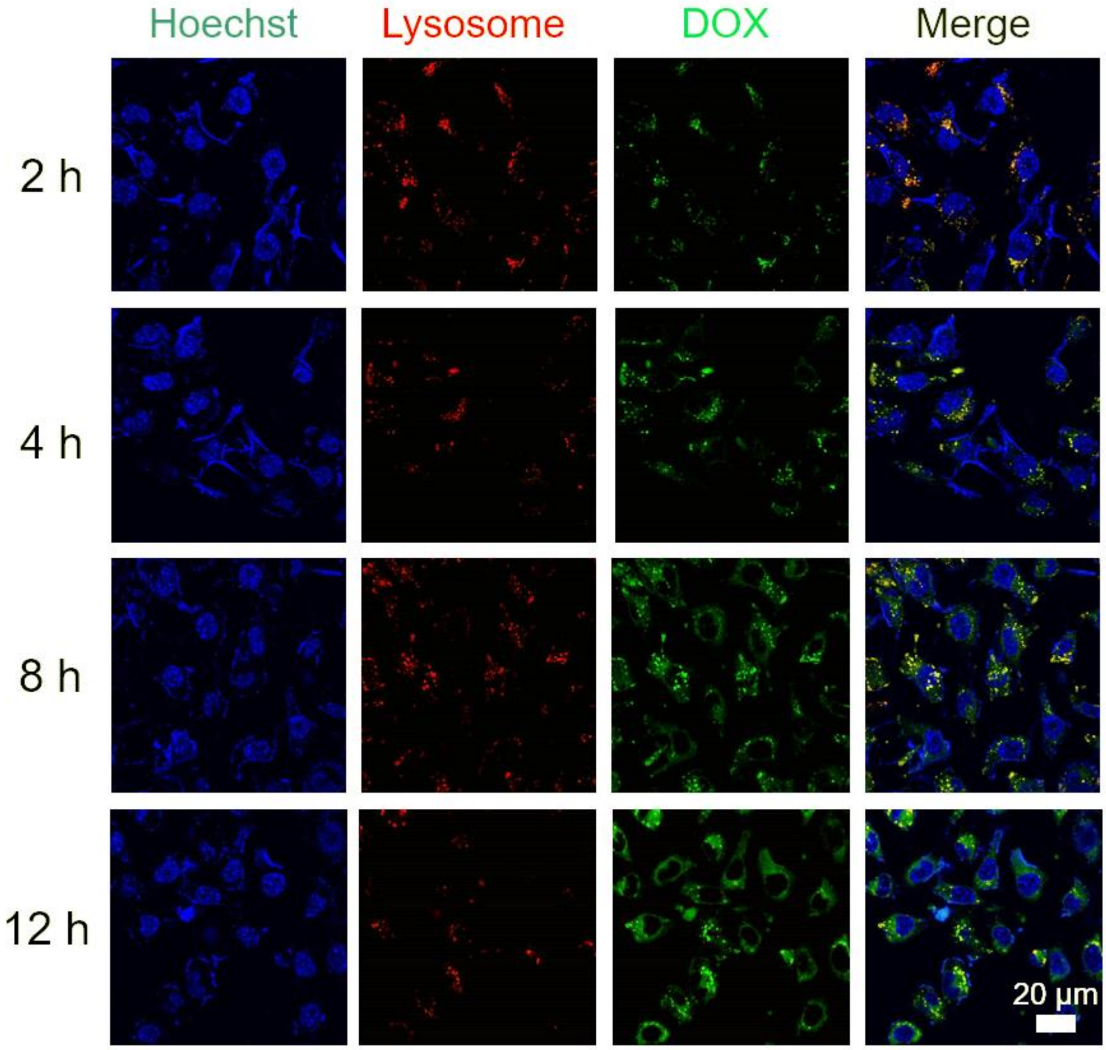

Figure S5. CLSM images of 4T1 cells showing the time-dependent uptake and intracellular trafficking of HCG. Scale bars: 20 μm.

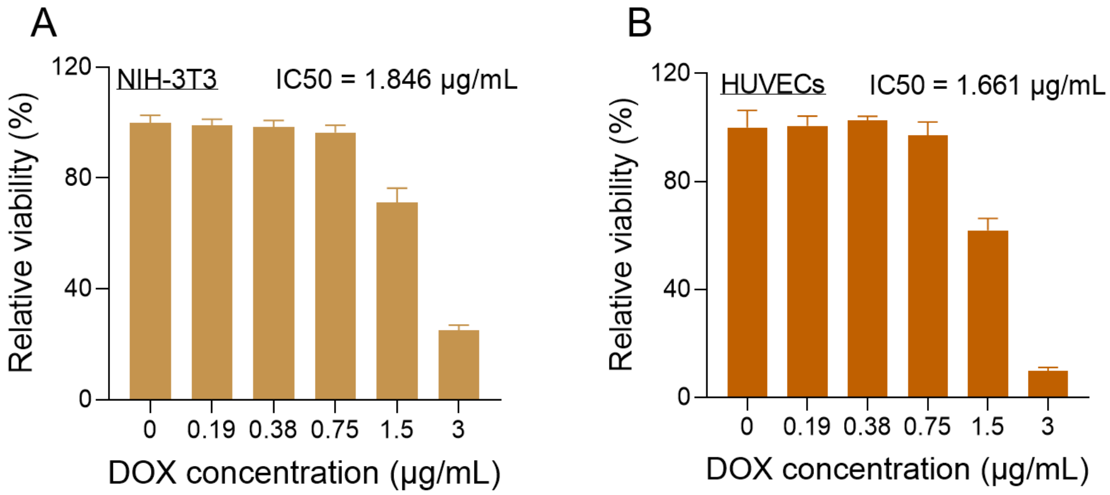

Figure S6. Cytotoxicity of different concentrations of HCG on (A) NIH-3T3 and (B) HUVECs.

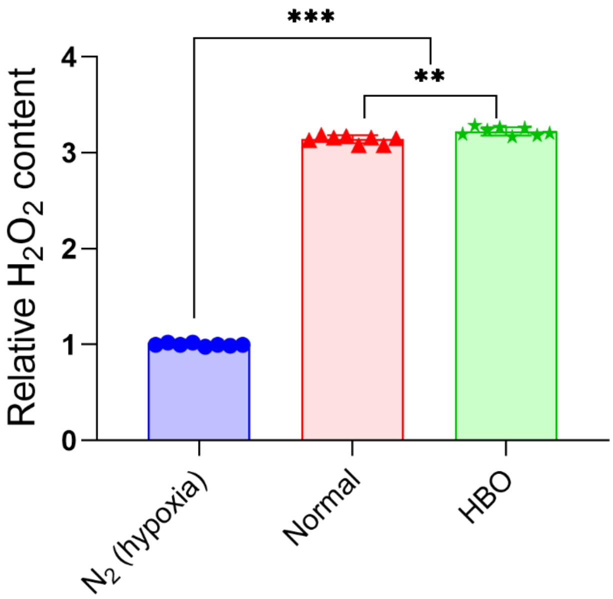

Figure S7. Relative content of H_2O_2 production catalyzed by GOD from glucose (5 mM) under different oxygen conditions. Statistical significance was calculated by t-test. *p* values: * p<0.05, ** p< 0.01, *** p < 0.001, ns stands for not significant.

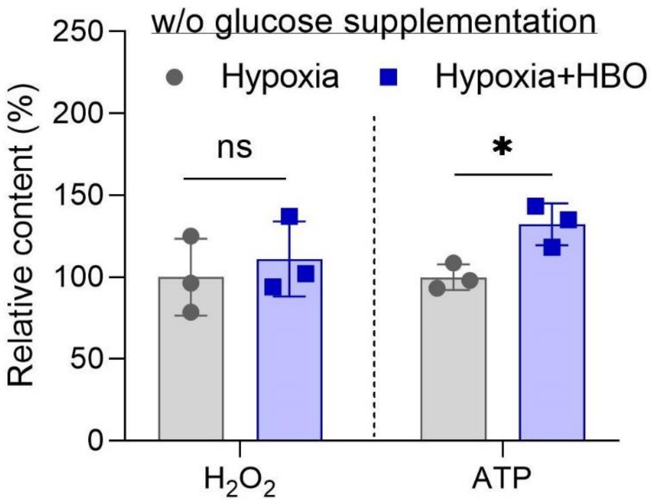

Figure S8. Relative H_2O_2 content, ATP level of HCG-treated 4T1 cells incubated in glucose-free medium.

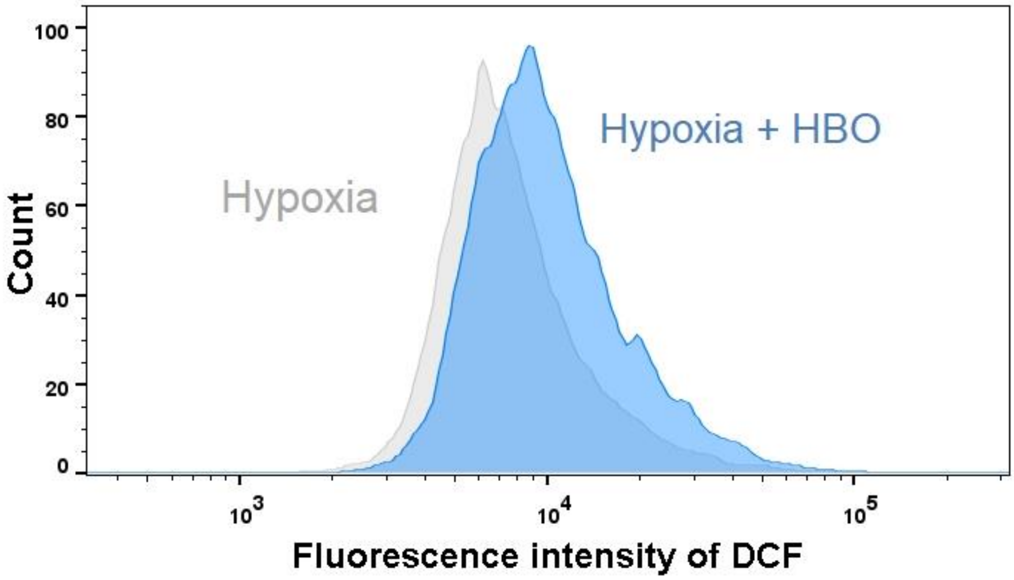

Figure S9. ROS level of HCG-treated 4T1 cancer cells incubated in hypoxia or hypoxia + HBO conditions.

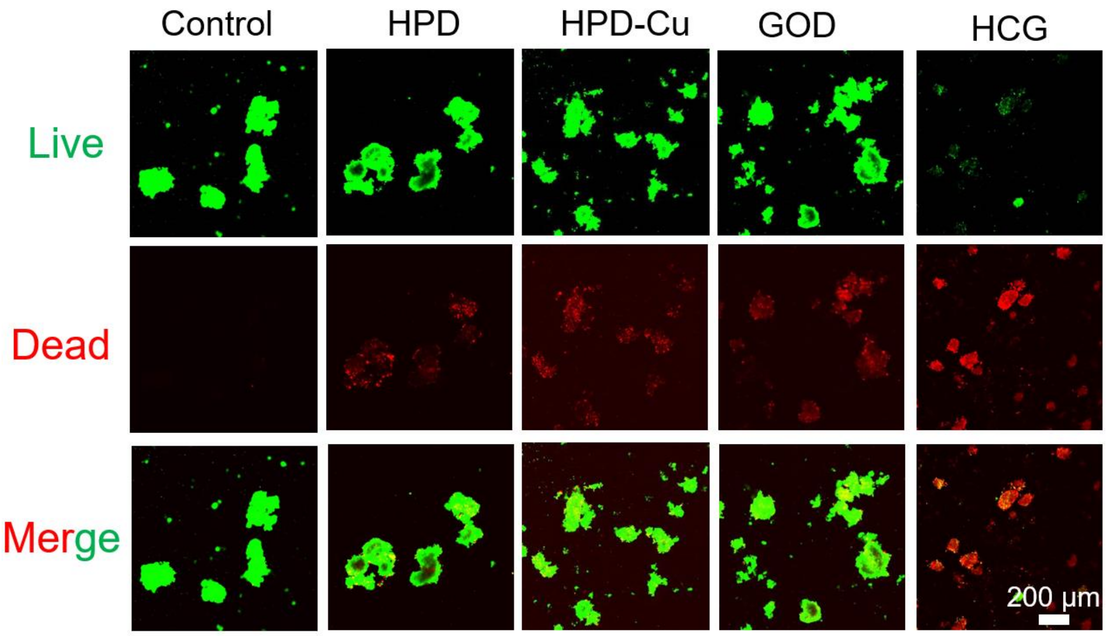

Figure S10. Live/dead co-staining of CSCs after different treatments. Scale bars: 200 μm.

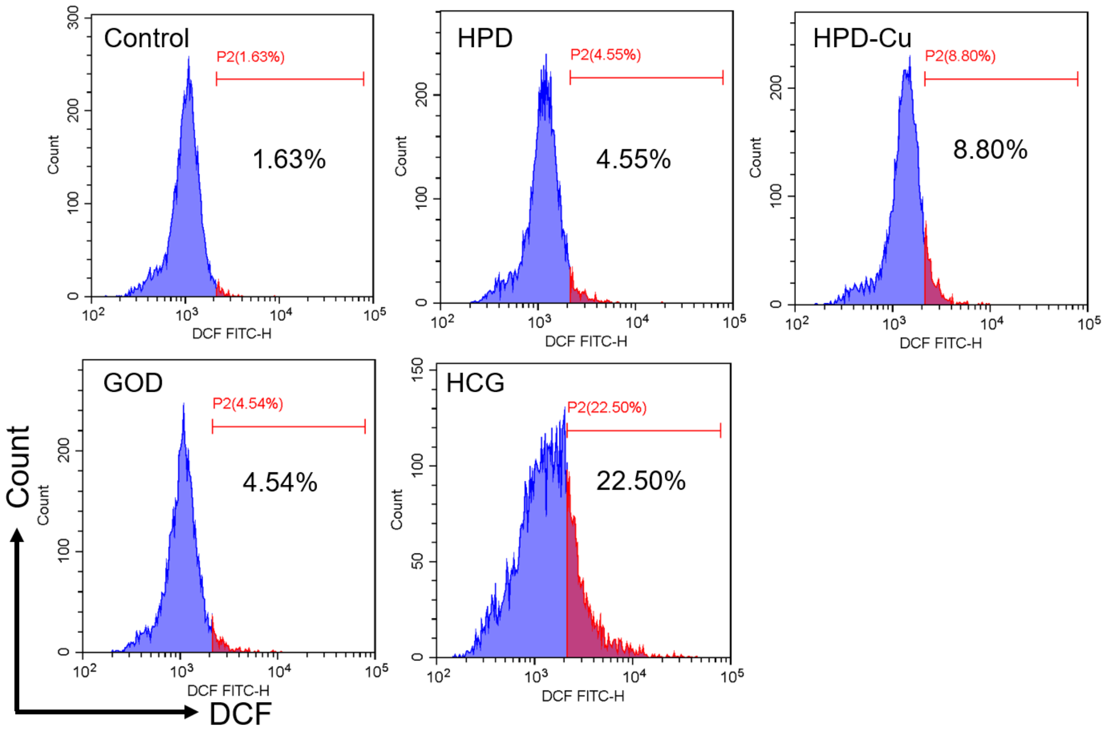

Figure S11. ROS level in CSCs after different treatments.

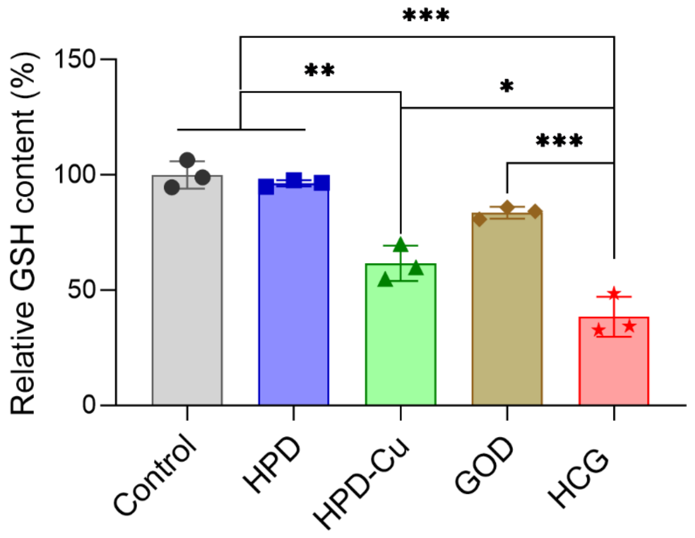

Figure S12. Relative GSH content in CSCs post various treatments. Statistical significance was calculated by t-test. p values: * p<0.05, ** p< 0.01, *** p < 0.001, ns stands for not significant.

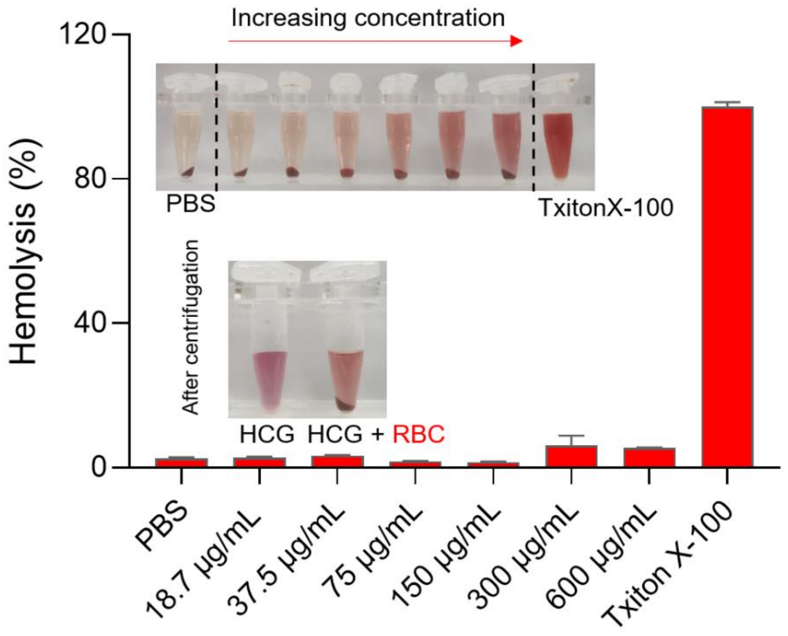

Figure S13. Hemolysis assay for different concentrations of HCG. Considering that HCG is difficult to be removed by centrifugation, we excluded the absorption of HCG itself when calculating the hemolysis rate.

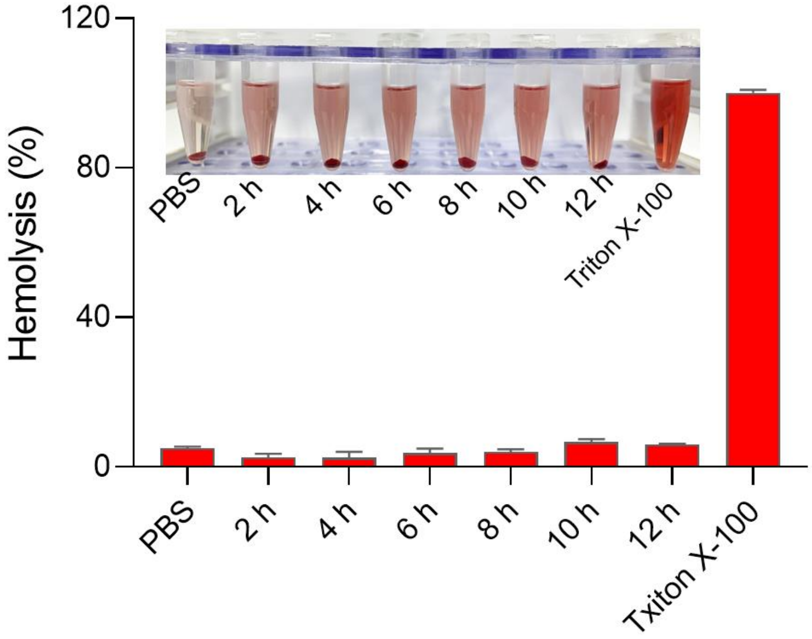

Figure S14. Hemolysis test of red blood cells after incubation with HCG for different times.

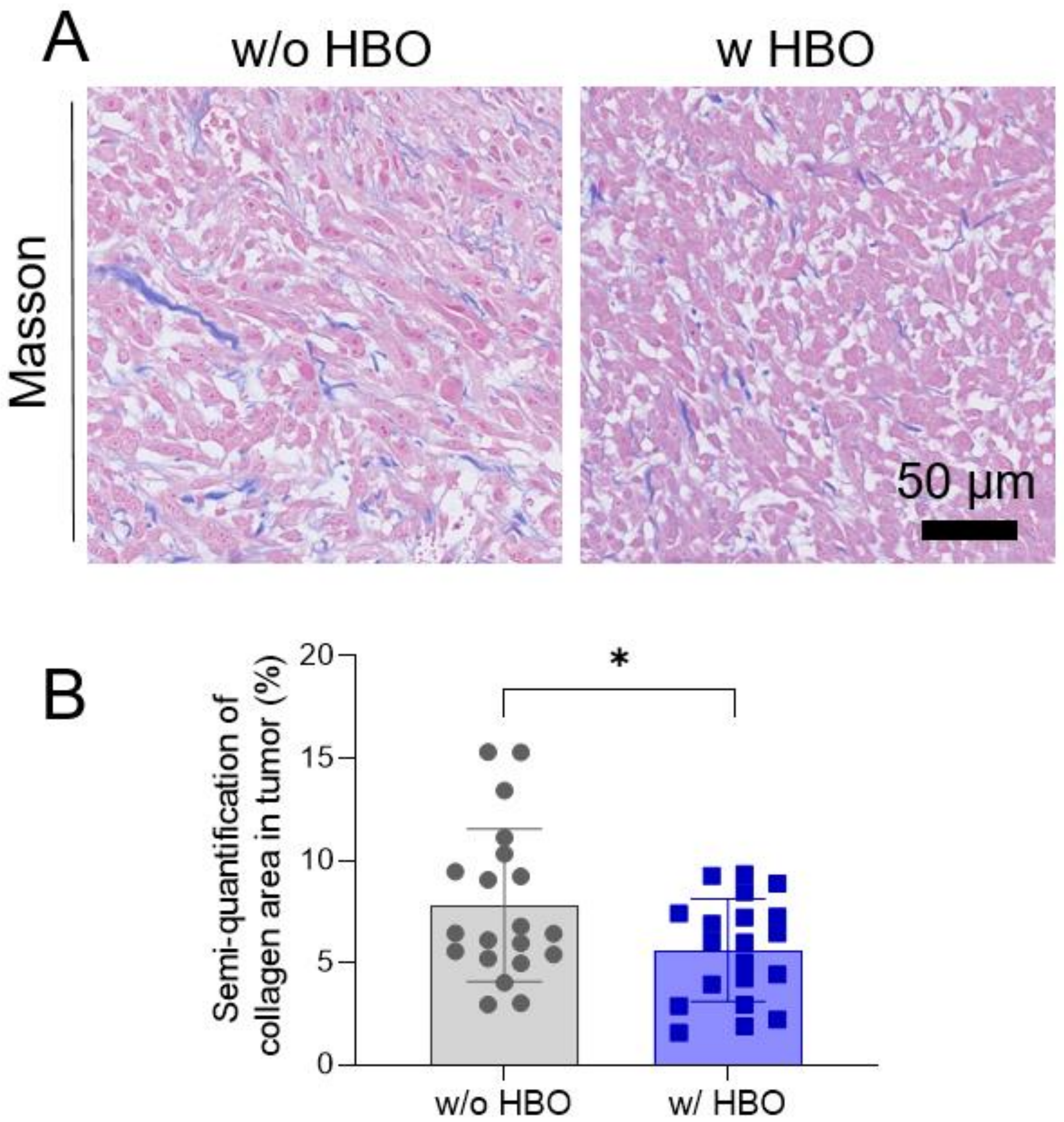

Figure S15. The impact of HBO on tumor tissue collagen. (A) Masson staining and (B) corresponding semi-quantitative results of tumor tissues with/without HBO treatment. Scale bars: 50 μm. Statistical significance was calculated by t-test. p values: * $p<0.05$, ** $p< 0.01$, *** $p < 0.001$, ns stands for not significant.

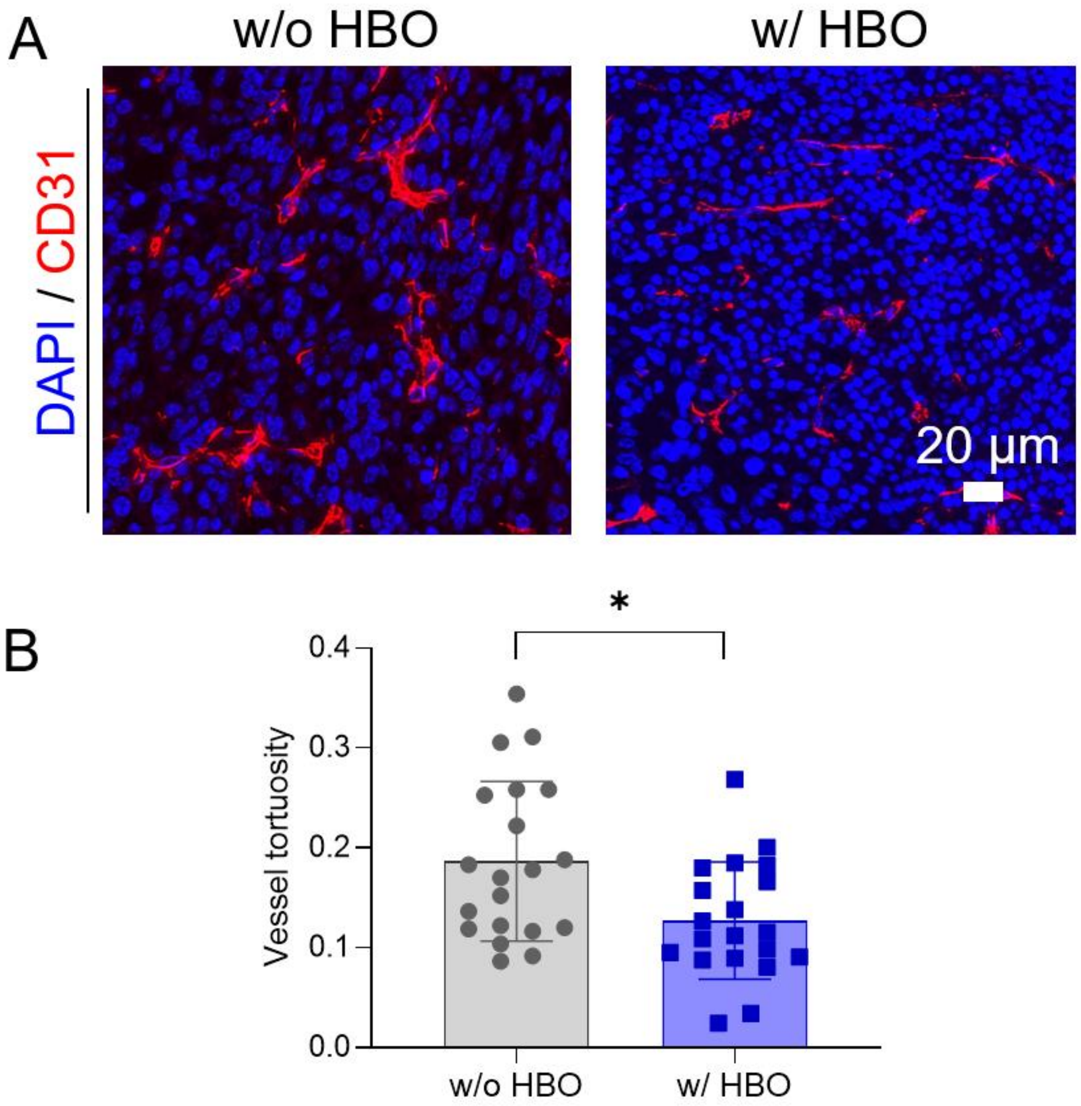

Figure S16. The effect of HBO on tumor blood vessel tortuosity. (A) CD31 staining and (B) corresponding semi-quantitative results of tumor tissues with/without HBO treatment. Scale bars: 20 µm. Statistical significance was calculated by t-test. p values: * $p<0.05$, ** $p< 0.01$, *** $p < 0.001$, ns stands for not significant.

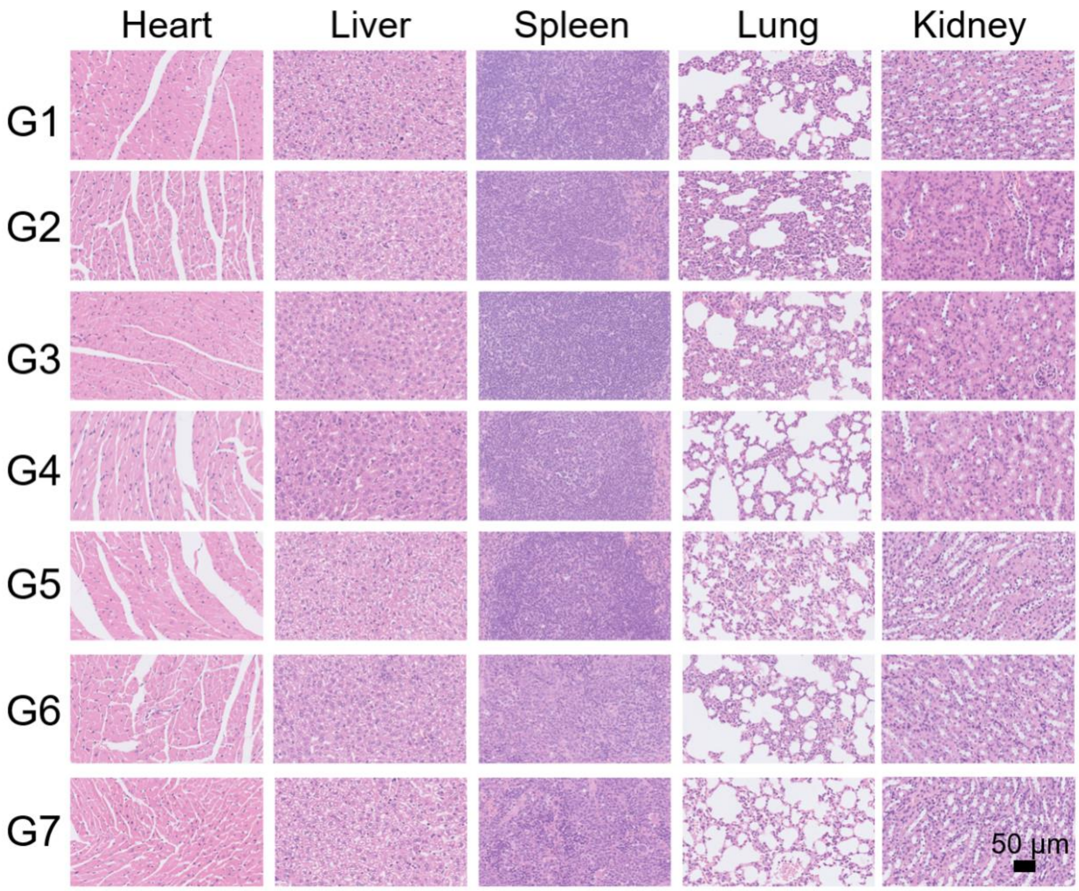

Figure S17. H&E staining of major organs after different treatments. G1: Control, G2: HBO, G3: GOD, G4: HPD, G5: HPD-Cu, G6: HCG, G7: HBO+HCG. Scale bars: 50 µm.

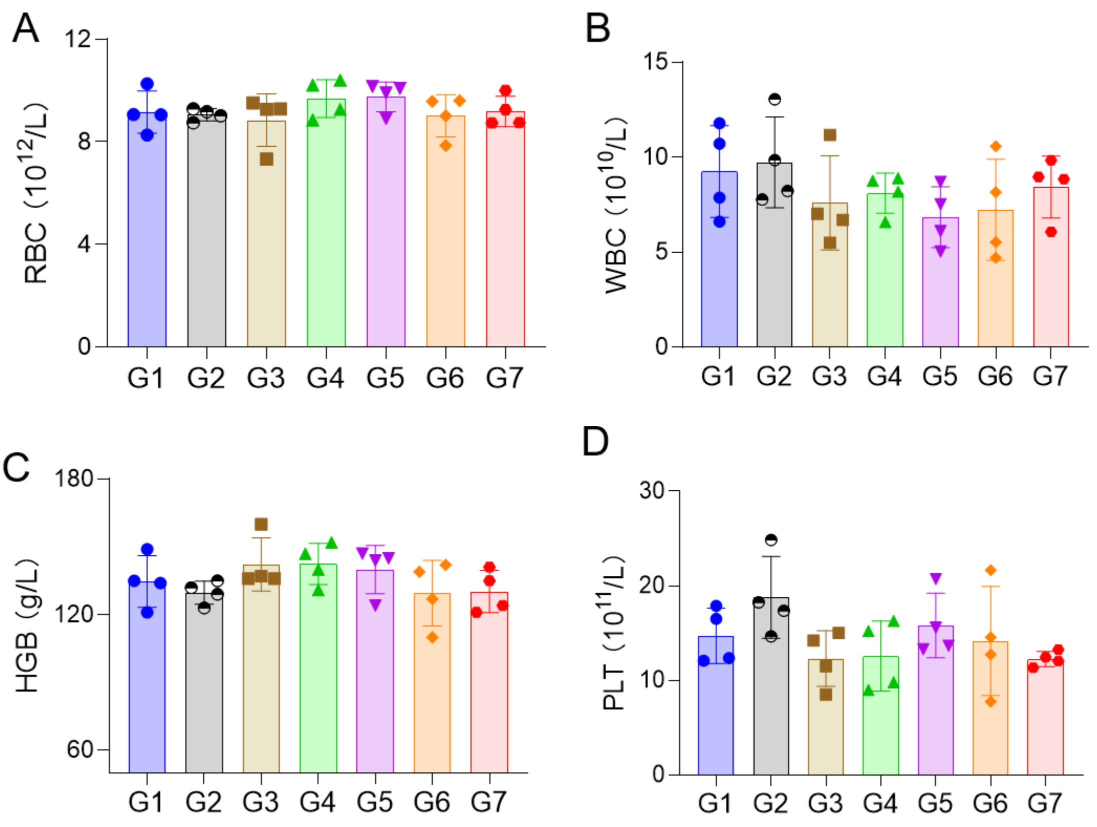

Figure S18. Routine blood analysis of mice in different treatment groups. G1: Control, G2: HBO, G3: GOD, G4: HPD, G5: HPD-Cu, G6: HCG, G7: HBO+HCG.

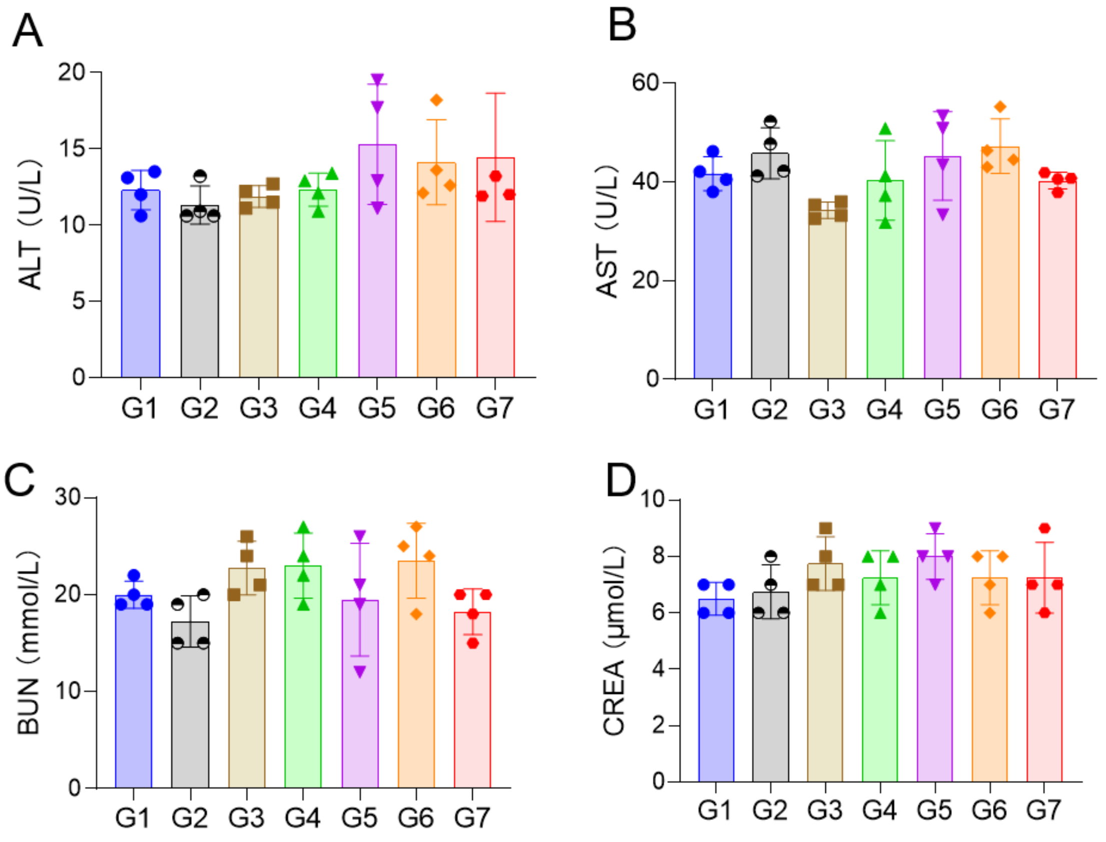

Figure S19. Blood biochemistry of mice after different treatments. G1: Control, G2: HBO, G3: GOD, G4: HPD, G5: HPD-Cu, G6: HCG, G7: HBO+HCG.

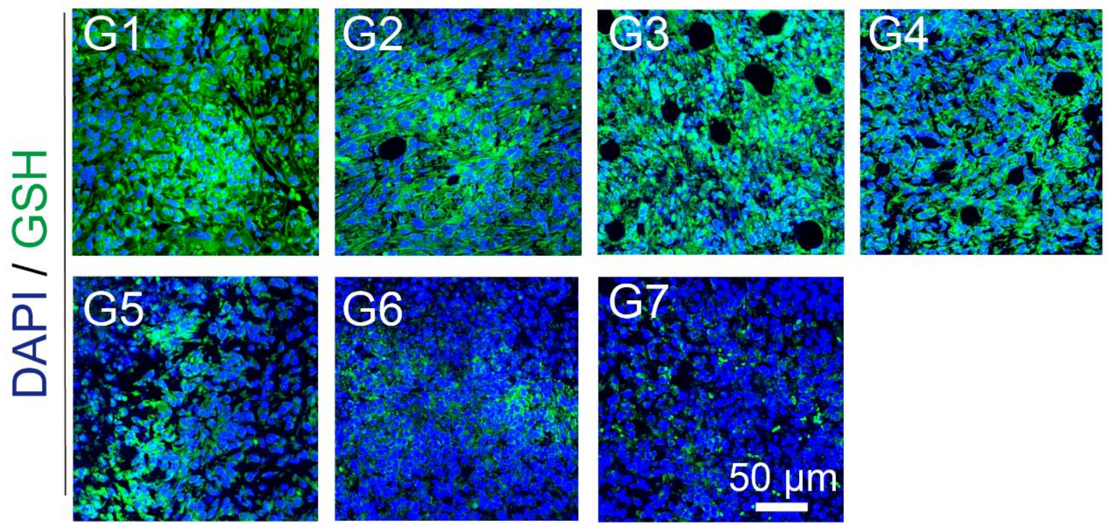

Figure S20. GSH staining of tumor tissues of different groups. G1: Control, G2: HBO, G3: GOD, G4: HPD, G5: HPD-Cu, G6: HCG, G7: HBO+HCG. Scale bars: 50 μm.

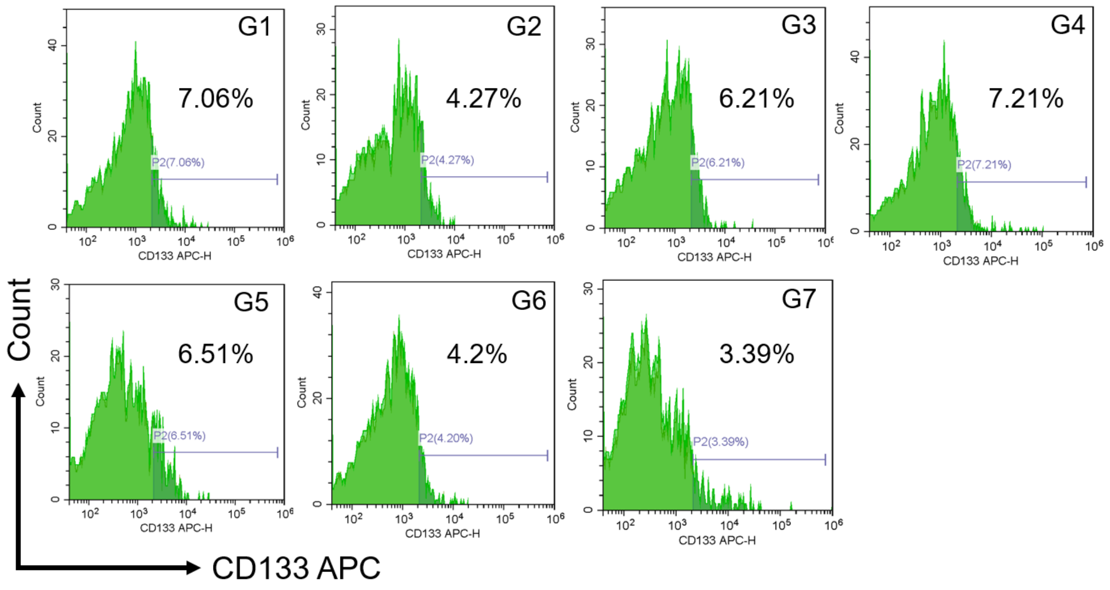

Figure S21. Clustering diagram of CD133^{+} CSCs in tumor tissues after different treatments. G1: Control, G2: HBO, G3: GOD, G4: HPD, G5: HPD-Cu, G6: HCG, G7: HBO+HCG.

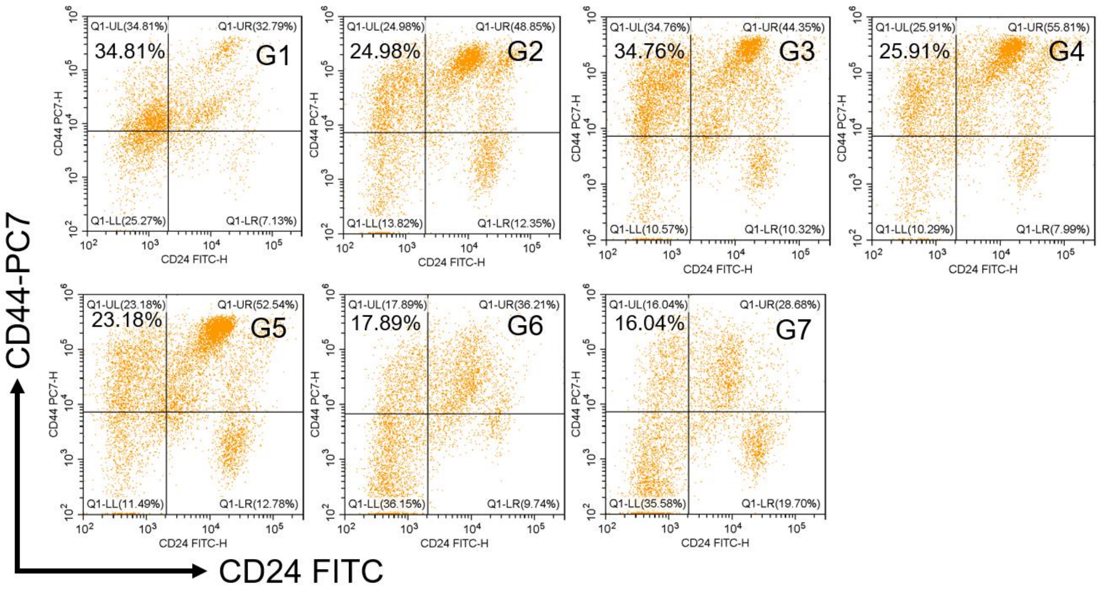

Figure S22. Clustering diagram of CD44^{+}CD24^{-} CSCs in tumor tissues after different treatments. G1: Control, G2: HBO, G3: GOD, G4: HPD, G5: HPD-Cu, G6: HCG, G7: HBO+HCG.

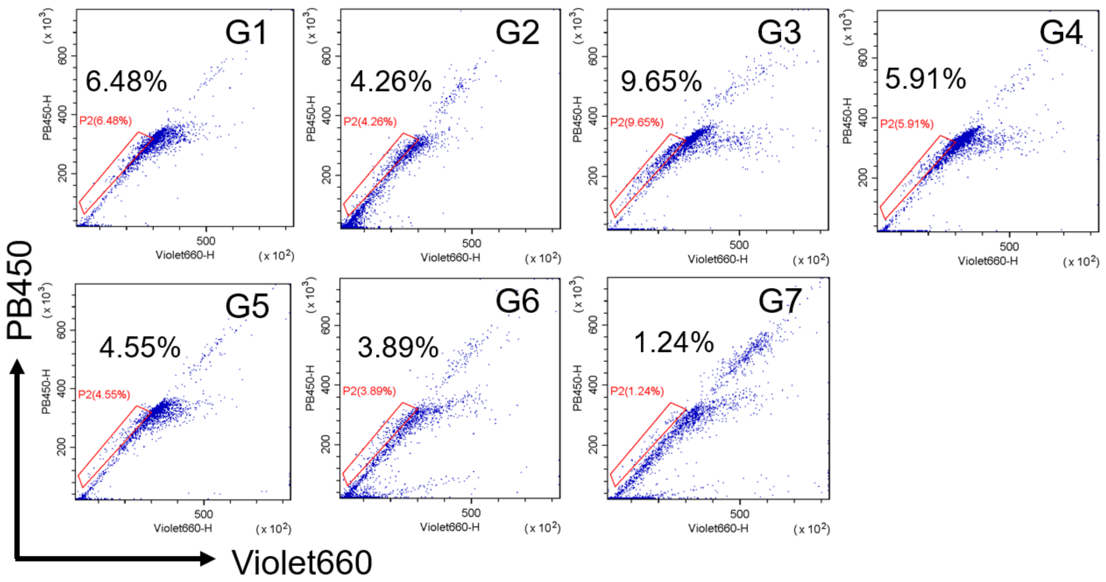

Figure S23. Clustering diagram of side population cells in tumor tissues after different treatments. G1: Control, G2: HBO, G3: GOD, G4: HPD, G5: HPD-Cu, G6: HCG, G7: HBO+HCG.

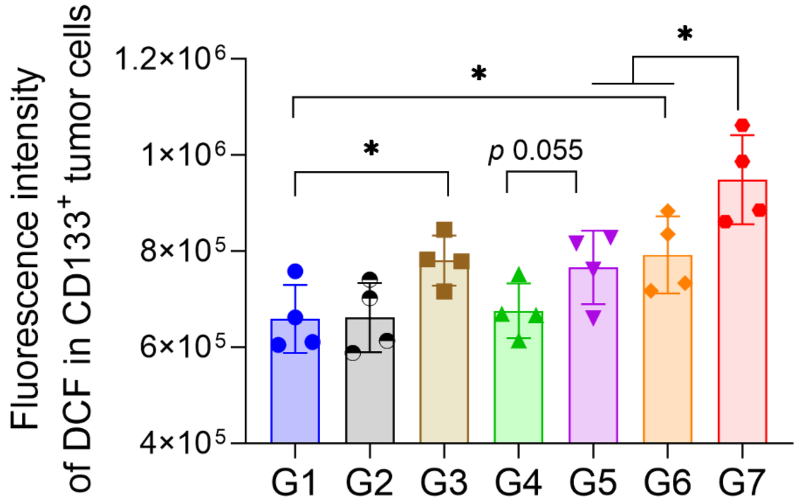

Figure S24. ROS levels in CSCs after different treatments measured by flow cytometry. G1: Control, G2: HBO, G3: GOD, G4: HPD, G5: HPD-Cu, G6: HCG, G7: HBO+HCG. Statistical significance was calculated by t-test. *p* values: * $p<0.05$, ** $p< 0.01$, *** $p < 0.001$, ns stands for not significant.

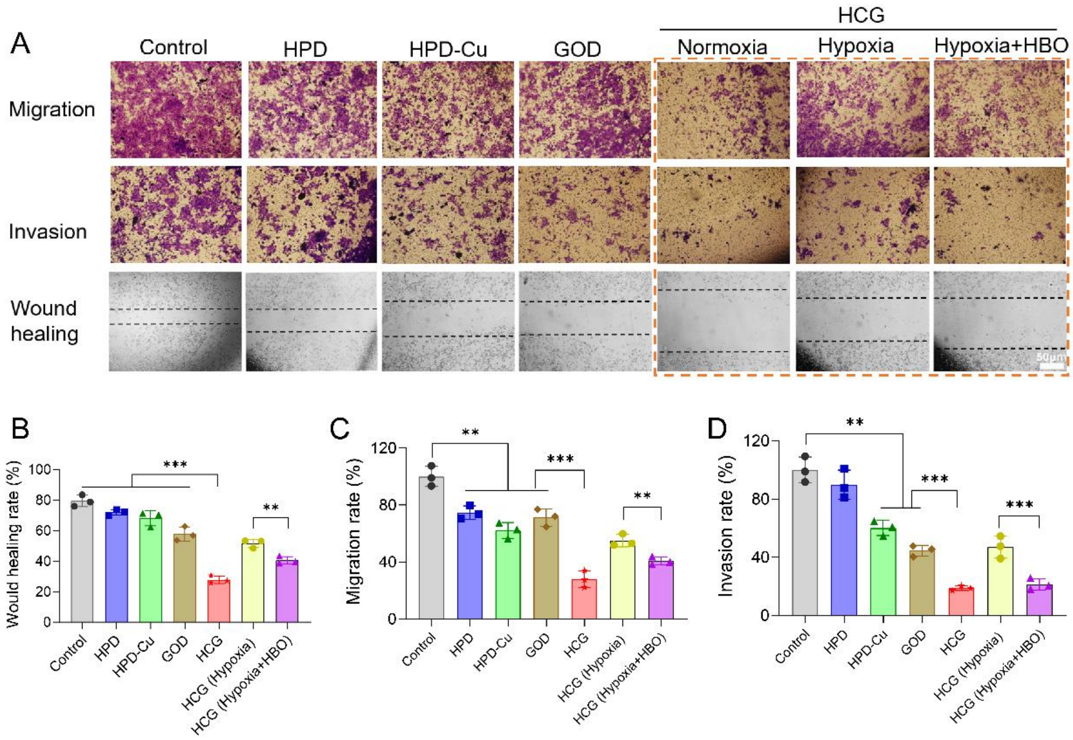

Figure S25. Anti-metastasis studies *in vitro*. (A) Microscopy images and quantitative analysis of the (B) wound healing and (C) migration as well as (D) invasion assays. Scale bars: 50 μm. Statistical significance was calculated by t-test. *p* values: * $p<0.05$, ** $p< 0.01$, *** $p < 0.001$, ns stands for not significant.

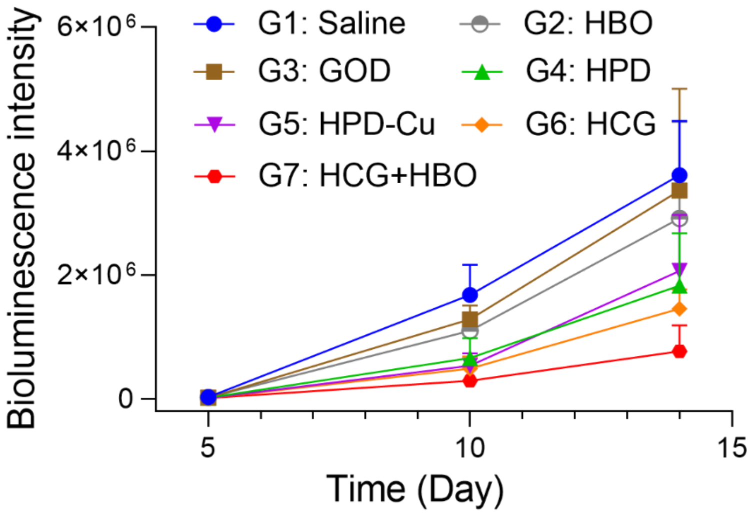

Figure S26. Mean bioluminescence intensity of lungs with different treatments.

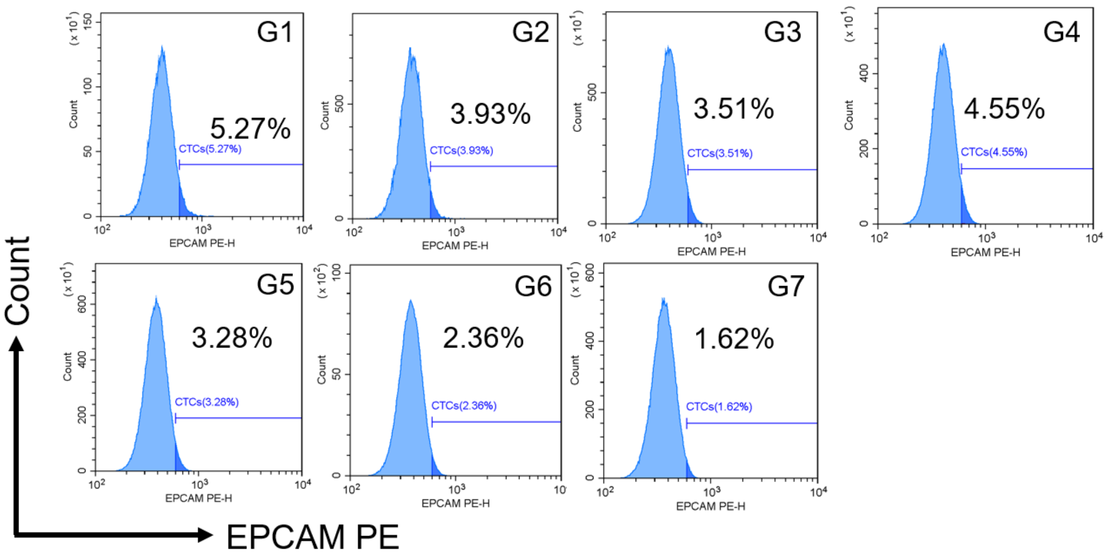

Figure S27. Percentage of circulating tumor cells (CTCs) in mice blood after different treatments. G1: Control, G2: HBO, G3: GOD, G4: HPD, G5: HPD-Cu, G6: HCG, G7: HBO+HCG.

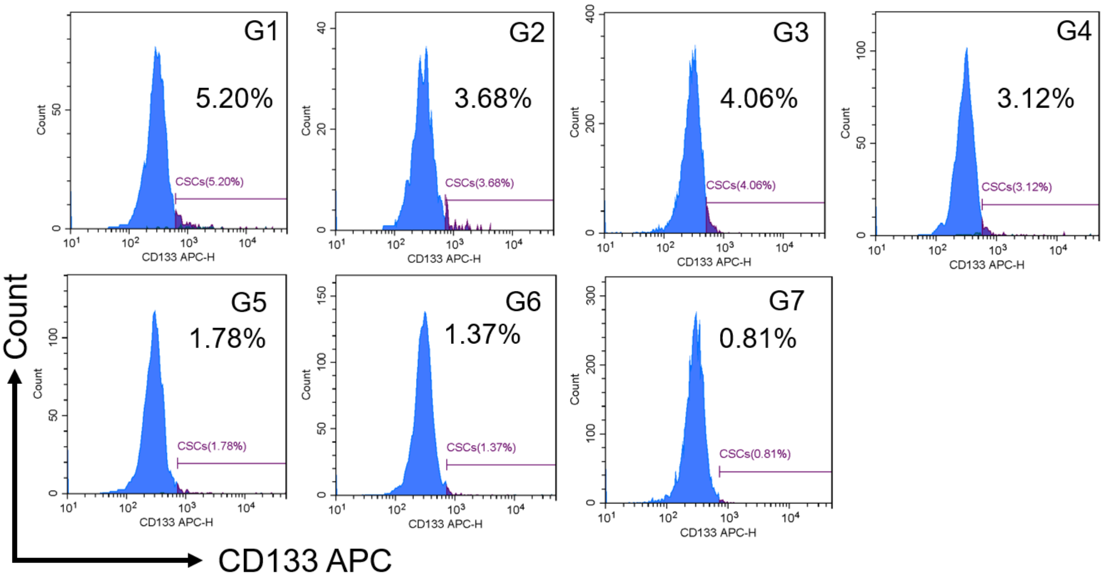

Figure S28. Percentage of $CD133^+$ CTCs in mice blood after different treatments. G1: Control, G2: HBO, G3: GOD, G4: HPD, G5: HPD-Cu, G6: HCG, G7: HBO+HCG.

Reference

[1] a) G. Dontu, W. M. Abdallah, J. M. Foley, K. W. Jackson, M. F. Clarke, M. J. Kawamura, M. S. Wicha, *Genes Dev* **2003**, *17*, 1253; b) X. Liu, N. Ye, C. Xiao, X. Wang, S. Li, Y. Deng, X. Yang, Z. Li, X. Yang, *Nano Today* **2021**, *40*, 101248.

[2] J. Liu, Y. Tan, H. Zhang, Y. Zhang, P. Xu, J. Chen, Y. C. Poh, K. Tang, N. Wang, B. Huang, *Nat Mater.* **2012**, *11*, 734.

[3] H. Hu, Y. Li, Q. Zhou, Y. Ao, C. Yu, Y. Wan, H. Xu, Z. Li, X. Yang, *ACS Appl Mater Interfaces.* **2016**, *8*, 30833.
